# Biochar Addition Altered Bacterial Community and Improved Photosynthetic Rate of Seagrass: A Mesocosm Study of Seagrass *Thalassia hemprichii*

**DOI:** 10.3389/fmicb.2021.783334

**Published:** 2021-12-02

**Authors:** Jian Zhang, Juan Ling, Weiguo Zhou, Wenqian Zhang, Fangfang Yang, Zhangliang Wei, Qingsong Yang, Ying Zhang, Junde Dong

**Affiliations:** ^1^CAS Key Laboratory of Tropical Marine Bio-Resources and Ecology, Guangdong Provincial Key Laboratory of Applied Marine Biology, South China Sea Institute of Oceanology, Chinese Academy of Sciences, Guangzhou, China; ^2^Southern Marine Science and Engineering Guangdong Laboratory (Guangzhou), Guangzhou, China; ^3^Key Laboratory of Tropical Marine Biotechnology of Hainan Province, Sanya Institute of Oceanology, South China Sea Institute of Oceanology, Chinese Academy of Sciences, Sanya, China; ^4^Innovation Academy of South China Sea Ecology and Environmental Engineering, Chinese Academy of Sciences, Guangzhou, China; ^5^College of Marine Science, University of Chinese Academy of Sciences, Beijing, China; ^6^Sanya National Marine Ecosystem Research Station, Tropical Marine Biological Research Station in Hainan, Chinese Academy of Sciences, Sanya, China

**Keywords:** seagrass, biochar addition, nutrient cycling, bacterial community, community assembly

## Abstract

Seagrass meadows, as typical “blue carbon” ecosystems, play critical ecological roles in the marine ecosystem and decline every year. The application of biochar in soil has been proposed as a potential soil amendment to improve soil quality and mitigate global climate change. The effects of biochar on soil bacterial activities are integrally linked to the potential of biochar in achieving these benefits. However, biochar has been rarely applied in marine ecosystems. Whether the application of biochar could work on the seagrass ecosystem remained unknown. In this study, we investigated the responses of sediment and rhizosphere bacterial communities of seagrass *Thalassia hemprichii* to the biochar addition derived from maize at ratios of 5% by dry weight in the soil during a one-month incubation. Results indicated that the biochar addition significantly changed the sedimental environment with increasing pH, total phosphorus, and total kalium while total nitrogen decreased. Biochar addition significantly altered both the rhizosphere and sediment bacterial community compositions. The significant changes in rhizosphere bacterial community composition occurred after 30days of incubation, while the significant variations in sediment bacterial community composition distinctly delayed than in sediment occurred on the 14th day. Biochar application improved nitrification and denitrification, which may accelerate nitrogen cycling. As a stabilizer to communities, biochar addition decreased the importance of deterministic selection in sediment and changed the bacterial co-occurrence pattern. The biochar addition may promote seagrass photosynthesis and growth by altering the bacterial community compositions and improving nutrient circulation in the seagrass ecosystem, contributing to the seagrass health improvement. This study provided a theoretical basis for applying biochar to the seagrass ecosystem and shed light on the feasible application of biochar in the marine ecosystem.

**Graphical Abstract:**
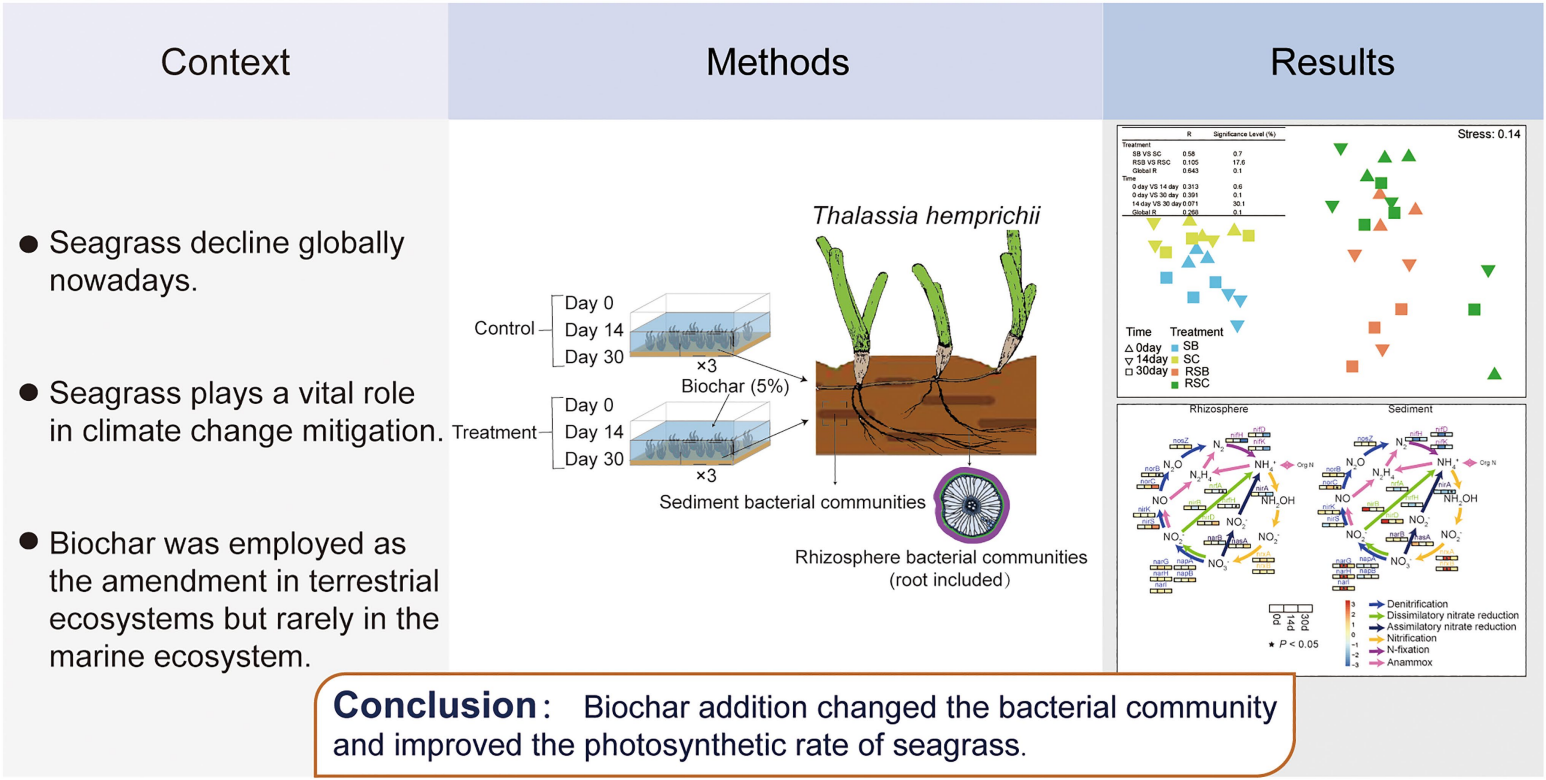

## Introduction

Seagrasses are the only marine flowering plants found along temperate and tropical coastlines worldwide, and seagrass meadows are one of the most widespread coastal habitats on earth (Cullen-Unsworth and Unsworth, 2018). Seagrass meadows play critical ecological roles in the marine ecosystem (Cullen-Unsworth and Unsworth, 2018). However, like many of the world’s natural habitats, seagrass meadows are in decline, with estimated global losses of ~7% annually since 1990 (Waycott et al., 2009). Poor coastal water quality and coastal development are among the primary drivers of their loss (Waycott et al., 2009). Anthropogenic pollution and global climate change altered the sedimental environment and nutrient cycles of the seagrass ecosystem (Short and Neckles, 1999; Harris et al., 2021). Strategies need to be implemented to relieve the pressure on seagrass.

Biochar is a carbon-rich coproduct resulting from pyrolyzing biomass in oxygen-limited conditions (Lee et al., 2010). It is also a stable carbonaceous material with an extensive surface area and active functional groups (Spokas, 2010). The biochar application in the soil is evaluated globally to improve soil fertility (Novak et al., 2009). Recently, researchers have shown an increased interest in biochar amendments because it could promote additional photosynthetically fixed carbon into the soil, where it may contribute to longer-term carbon storage and thus mitigates increasing atmospheric CO_2_ concentrations (Schmidt and Noack, 2000; Lehmann et al., 2006; Lehmann, 2007; Woolf et al., 2010). It is generally accepted that biochar is mainly unavailable to soil microbes, but it can induce changes in soil physicochemical properties and the introduction of metabolically available labile carbon compounds associated with the biochar, which may shift the soil microbial community composition and abundance (Grossman et al., 2010; Anderson et al., 2011). The variations mentioned above may well affect nutrient cycles or soil structure and indirectly affect plant growth (Yu et al., 2021).

Sediment, especially the rhizosphere of plants, is a complex and heterogeneous hotspot inhabited by various microorganisms, including bacteria, fungi, protists, nematodes, and viruses (Dodd et al., 1987; Huang et al., 2014; Wei et al., 2017; Pratama et al., 2020). Plants provide a multitude of niches for microorganisms’ growth and proliferation. Lennon and Jones (2011) noted that the physicochemical properties of the soil, together with plant species, dominated where members of microorganisms can grow and thrive. Vice versa, soil microbial communities play central roles in most biogeochemical and ecological processes (Bardgett and van der Putten, 2014). They can form complex co-associations with plants and have essential roles in promoting the productivity and health of the plant in natural environments (Trivedi et al., 2020). Among the plant-associated microbiota, bacteria are the most dominant form. Thus, information on bacteria community composition, diversity, and their determinants is critical for understanding responses of plant microbial symbiont to environmental changes. Moreover, Martin et al. (2020) found that microbial indicators could detect the potential stress in the seagrass ecosystem while other seagrass health metrics failed to detect.

So far, biochar has been widely studied in terrestrial ecosystems (Laird et al., 2010; Song et al., 2020; Owsianiak et al., 2021), and biochar application in soil has been proven to be an effective method for enhancing nutrient cycling, and they could mediate biochar-plant root interactions and ultimately affected root growth and overall plant performance (El-Naggar et al., 2019; Purakayastha et al., 2019). Zhang et al. (2018) found that the soil microbial activities increased and community structure changed under biochar amendment, which benefited the soil carbon sequestration and farmland systems stability and promoted soil nutrients cycling, thus improving crop yields. Plant stress is one of the major problems encountered in plant growth, and Kavitha et al. (2018) found biochar displayed great potential to mitigate plant stresses for both biotic and abiotic types of stresses. However, biochar application was mainly investigated in terrestrial agriculture and freshwater ecosystems, while for the marine environment, the information was rare. Given the functions of biochar to the terrestrial ecosystems, whether biochar could be applied to the marine seagrass ecosystem and get similar results. If biochar could promote the seagrass ecosystem, such as optimization of nutrient cycles and promotion of seagrass growth, it may be a solution to mitigate seagrass stresses under anthropogenic activities and global climate change.

In the present study, biochar was added as a soil amendment with the intention to improve the health condition of seagrass. The high-throughput DNA gene sequencing has been used to investigate the influence of biochar on the bacterial community, which indirectly explains biochar’s effect on seagrass. The purpose of this study was to synthesize responses of seagrass sediment and rhizosphere bacterial community structure shifts and activities to biochar addition comprehensively. We have a hypothesis that if biochar addition could optimize the sediment and rhizosphere bacterial community structure of seagrass in the marine ecosystem and indirectly ameliorate the health status of seagrass? The result of this study could contribute to further biochar application in the seagrass ecosystem.

## Materials and Methods

### Sample Collection, Microcosm Setup, and Experimental Design

Biochar was pyrolyzed from maize straw in this study. The maize straw was firstly pre-crushed, dried at 80°C, passed through a 2-mm sieve, and then pyrolyzed at 600°C for 1h in the oven. Biochar pH was 9.0 approximately, which was measured with the standard procedure referred to ASTM (2017).

Seagrass *Thalassia hemprichii* were collected at Xincun Bay, Hainan province, China, (18°24′48″ N, 109°59′2″ E) on 13th June 2018. The *in situ* sediment was collected, extracting the surface layer (up to 10cm deep) simultaneously. The samples were collected, stored in sterile sealing bags, and immediately transported to the laboratory.

The culture experiment was conducted indoors with constant room temperature at Tropical Marine Biological Research Station in Hainan, Chinese Academy of Sciences, from June 13, to July 19, 2018. Six independent microcosms manufactured by rectangular glass aquaria (24L capacity, 30cm height×40cm length×20cm width) were used for the experiment. Each microcosm contained about 10cm of sediment (about 10kg wet weight) and 10L of artificial seawater, configured according to the ambient salinity (28.2 PSU) in the lab. Seagrasses were then transported into the glass vessels, where they were maintained for 1week of indoor acclimation.

After the acclimation period, three aquaria with non-biochar-added soil were set as the blank control groups, while three aquaria with biochar-added soil with a final concentration of 5% were set as biochar addition groups. Each aquarium had an independent air pump providing proper aeration. The temperature was maintained at 29.0°C with a slight fluctuation (±0.5°C), close to the ambient temperature at the collection site (29.5°C). The lab allowed us to control incident light (270μmol photons m^−2^ s^−1^) above the saturation irradiance for these plants (Pérez and Romero, 1992) on a 12-h:12-h light: dark photoperiod. In order to better mimic the environmental conditions and eliminate artificial disturbances, no extra nutrients were added to the samples during the experiments, and the seawater overlying sediment was renewed every week with 0.2μm membrane filtered seawater.

Samples used for bacterial and physicochemical analysis were collected simultaneously on the 1st, 14th, and 30th days. Sediment samples at each aquarium were collected from unvegetated areas. Rhizosphere sediment samples include the root of seagrass and soil that adheres to roots. After sampling, each sample was thoroughly homogenized using a sterilized spoon. All samples consisted of four types, including the rhizosphere sediment of blank control (RSC), the sediment of blank control (SC), the rhizosphere sediment of experiment group (RSB), and the sediment of experiment group (SB). “Soil” refers to both the sediment and rhizosphere sediment in afterward description.

All samples for DNA analysis were kept in sample protectors (TaKaRa, Dalian, China), frozen immediately, and stored at −80°C until further analysis. The temperature and salinity of the seawater adjacent to seagrass samples (within 3cm) were measured using a YSI 6600V2 water quality sonde (YSI, Yellow Springs, OH, United States). Dissolved oxygen (DO) concentrations and pH values were measured using a portable pH/DO Meter (Thermo Fisher Scientific, MA, United States). Inorganic nutrients in seawater, including ammonium (oxidized by hypobromite), nitrate (diazotizing with sulfanilamide), nitrite [colored N-(1-naphthyl)-ethylenediamine-dihydrochloride], and phosphate (colored molybdophosphoric blue), were measured using standard methods as described previously with spectrophotometer (Huang et al., 2003). Chemical data [Total nitrogen (TN), total phosphorus (TP), total kalium (TK), available nitrogen, available phosphorus, available kalium, and nitrate-nitrogen (NO3-N)] of sediments were determined by using standard oceanographic methods with ultraviolet spectrophotometry method (General Administration of Quality Supervision, Inspection and Quarantine of the People’s Republic of China, 2002). The rapid light curve (RLC) function of the Diving-PAM (Diving-PAM, Walz, Germany) was used to measure *in situ* photosynthetic performance (based on the effective quantum yield of PSII [Y] values) of intact seagrasses that were placed in small incubating chambers, and the rate of electron transport between photosystem II and photosystem I (ETR) was measured and used as a proxy for the photosynthetic rate.

### DNA Extraction, PCR, and Sequencing

The bacterial 16S rRNA gene (total bacterial composition) was amplified using universal 16S rRNA gene (V4-V5) primers 515F-Y (5′-GTGYCAGCMGCCGCGGTAA) and 926R (5′-CCGYCAATTYMTTTRAGTTT). PCR cycling was performed in reaction mixtures consisting of 25μl Ex Taq (2×; TaKaRa, Dalian, China), 1μl of forward primer (10μm), 1μl of reverse primer (10μm), and 1μl of DNA in a 50-μl final volume. The PCR amplification program was as follows: initial denaturation at 94°C for 5min, followed by 35cycles of denaturation at 94°C for 30s, annealing at 54°C for 45s and extension at 72°C for 45s, and final elongation at 72°C for 10min. Libraries were constructed from the purified PCR products of each sample. The DNA was then purified with a Promega Wizard DNA Clean-Up System (Madison, WI, United States). Sequencing was performed on the Illumina MiSeq platform 2×250bp.

Amplicon bioinformatic analysis was accomplished with EasyAmplicon v1.0 (Liu et al., 2021). Paired-end sequence data were merged, quality filtered, and dereplication using VSEARCH v2.15 subcommand –fastq_mergepairs, −fastx_filter and –derep_fulllength, respectively (Rognes et al., 2016). Then, the non-redundancy sequences are denoising into amplicon sequence variants (ASV) with USEARCH v10.0 (Edgar, 2010; *via* unoise3), and then, the singletons and chimeric sequences were removed. Chimera was removed by VSEARCH –uchime_ref against with SILVA database (Quast et al., 2013). Feature tables were created by vsearch –usearch_global. The USEARCH sintax algorithm classified the taxonomy of the features (ASVs) in RDP training set 16 (Cole et al., 2014). Samples were rarefied to 10,392 sequences per sample. The soil microbiome data set has been deposited in the NCBI Sequence Read Archive under accession number PRJNA750881.

### PICRUSt2

Functional predictions of the microbial community were conducted using Phylogenetic Investigation of Communities by Reconstruction of Unobserved States 2 (PICRUSt2) and the default analysis parameters (Douglas et al., 2020). PICRUSt2 uses the following tools and algorithms: HHMER, EPA-NG, GAPPA, and castor to align ASVs to reference sequences, place them into a reference tree, and perform hidden-state prediction functions (Eddy, 1998; Louca and Doebeli, 2018; Barbera et al., 2019; Czech et al., 2020; Douglas et al., 2020), respectively. Functional prediction analysis was performed at the gene-level (KEGG orthologs) and the pathway-level (Meta Cyc; Kanehisa and Goto, 2000; Karp et al., 2002). The nearest sequenced taxon index (NSTI) value is calculated to evaluate the prediction accuracy, and lower value means higher accuracy. In this study, NSTI values were 0.15±0.002 (mean±s.e., *n*=39). The gene table was compared with KEGG pathways related to nitrogen metabolism (KO00910). As a result, a total of 18 nitrogen cycling genes (KOs) were chosen for subsequent analyses. The details of these genes (KOs) were shown in Supplementary Table S2. Furthermore, 30 genes involved in the carbon fixation, phosphorous, and sulfur metabolism were also selected for subsequent analyses.

### Statistics Analysis

The phylogenetic diversity index (alpha diversity) and rarefaction curves were calculated based on the rarefied ASV table using the “vegan” R package in R software (version 4.0.4; Oksanen et al., 2015). All heatmap was generated using the “pheatmap” package in the R environment (R Core Team, 2018). The correlation between environmental variables and community composition was calculated using the “ggClusterNet”^1^ R package in R software (version 4.0.4) with mantel test. Statistical analysis of metagenomic profiles (STAMP) was conducted to analyze the abundance profile. A two-sided Welch’s *t* test carried by STAMP was used to identify distinct taxonomic compositions and metabolic pathways between blank control and experiment group (Parks et al., 2014; Li et al., 2020a).

To compare the β diversity of communities, non-metric multidimensional scaling ordination (nMDS) analyses were conducted based on Bray-Curtis similarity. Furthermore, an analysis of similarity (ANOSIM) was used to statistically test for significant differences in bacteria communities among groups, based on different times and treatments. In this analysis, complete separation is indicted by *R*=1, whereas *R*=0 suggests no separation. Both nMDS and ANOSIM were performed in PRIMER 7.0 (Clarke and Gorley, 2015).

### Ecological Processes Influencing Bacterial Community Assembly

The null model (NM) was used to quantify the contributions of different ecological processes (stochastic vs. deterministic) to bacterial community structure (Stegen et al., 2013). The NM is pattern-generating model that deliberately exclude a mechanism of interest and allow for randomization tests of ecological and biogeographic data, a framework to quantitatively infer community assembly mechanisms by phylogenetic bin-based null model analysis (iCAMP) was used (Ning et al., 2020). We calculated the framework for bacterial community assembly in soil with the “iCAMP” R package^2^, and the results showed the relative importance of different processes in the turnover of each bin within each group of samples.

### Co-occurrence Network

A valid co-occurrence correlation was assigned between bacterial community composition if the spearman’s correlation coefficient (*r*) was greater than 0.6 with an adjusted value of *p*<0.01. Topological characteristics were calculated to describe the complexity of gene co-occurrence networks, including average degree (avgK, which is a key topological property to describe how well a node is connected to the others, higher avgK value means a more complex network), clustering coefficient (CC, which is used to measure the extent of module structure present in a network), characteristic path distance (CPD, which is the average value of the distances between every two nodes in a network, higher CPD value means a reduced coupling among nodes in a network), and network density (ND, which is closely related to the average degree).

The topological role of each ASV was determined according to the Zi degree (how well a node is connected to other nodes in the same module) and Pi degree (how well a node is connected to the nodes in other modules; Xun et al., 2017). According to the suggested Zi and Pi degree thresholds (Olesen et al., 2007), all ASVs were categorized into four subcategories: peripherals (Zi≤2.5 and 0≤Pi≤0.62), connectors (Zi≤2.5 and Pi>0.62), module hubs (Zi>2.5 and Pi≤0.62), and network hubs (Zi>2.5 and Pi>0.62). Overall, the correlations were calculated using the psych package (version 1.8.12; Revelle, 2017) in R software (version 4.0.4). The networks were visualized, and the topological characteristics were calculated using Gephi software (version 0.9.2; Bastian et al., 2009).

## Results

### Responses of the Environment to Biochar Addition

The environmental parameters of the water, sediment, and seagrass samples were shown in Supplementary Figure S1. The addition of biochar significantly increased sediment pH from 7.94±0.21 (mean±s.e.) to 8.31±0.08 (mean±s.e.). Total phosphorus, available phosphate, total kalium, and available kalium of sediment were also significantly increased with biochar addition on day 30 (*p*<0.05). Total nitrogen of the sediment was significantly decreased with biochar addition on day 30 (*p*<0.05). There was no significant difference in the final NO_3_-N concentration between control and biochar addition groups in sediment (Supplementary Figure S1). Biochar addition significantly increased the photosynthetic rate of seagrass from 0.76±0.006 (mean±s.e.) to 0.78±0.003 (mean±s.e.) on day 30.

### Community Structure and Diversity

Bacterial community profiling of 18 sediments (three replications for biochar addition and three replications for control at three time points) and 21 rhizosphere samples (three replications for biochar addition and four replications for control with three time points) were conducted to investigate the effects of biochar on the structure of bacterial communities. The bacterial community profiling yielded 405,288 high-quality sequences. A total of 7,357 bacterial ASVs were identified across all samples (Supplementary Table S1). For α-diversity analyses, the communities were rarified to 10,392 sequences per sample, which captured most of the observed ASV richness (Supplementary Figure S2). The sediment bacterial community profiling yielded 6,206 ASVs with 187,056 sequences, while the rhizosphere bacterial community got 7,198 ASVs with 218,232 sequences (Supplementary Table S1).

The Shannon index, providing an estimate of alpha diversity in each sample, ranged from 6.72 to 7.45 with a mean of 7.17±0.19 (mean±s.e.) and did not differ significantly between the control and treatment group (*p*>0.05; Supplementary Figure S3). Other alpha diversity indices (including ACE and Simpson) also did not show significant differences within and between groups (ANOVA, *p*>0.05).

**Figure 1:**
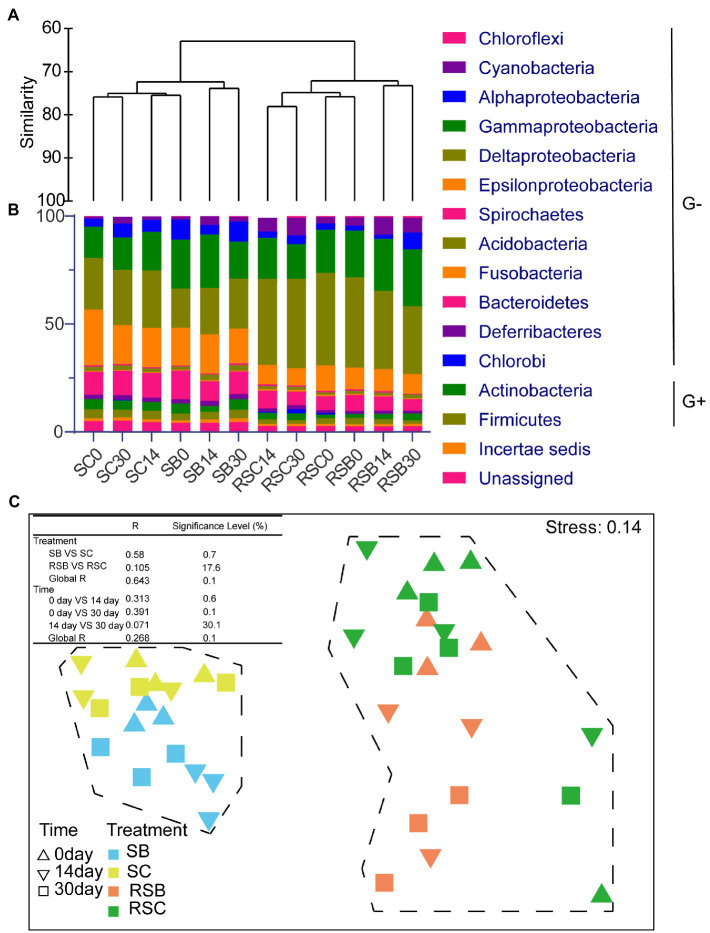
**(A)** Dendrogram cluster for 12 combined samples based on Bray-Curtis similarity. **(B)** Histogram showing the relative abundance of different subgroups (Phylum and Class level), G+ means Gram-positive bacteria while G− means Gram-negative bacteria. **(C)** Non-metric multidimensional scaling ordinations (nMDS) for bacteria communities of 39 samples. [Table in the figure were analysis of similarities (ANOSIM) of bacterial communities. SB, Sediment bacteria of treatment groups; SC, sediment bacteria of control groups; RSB, rhizosphere bacteria of treatment groups; RSC, rhizosphere bacteria of control groups].

Seagrass rhizosphere sediment and sediment presented different microbial habitats with specific bacteria (Supplementary Figure S4). The differences between bacterial communities (β-diversity) were visualized and quantified using the dendrogram cluster for 12 subgroups from the control and biochar addition groups based on Bray-Curtis similarity (replicates were combined into one subgroup; Figure 1A). Bacterial communities of rhizosphere sediment and sediment were clearly separated into two clusters. It revealed that there were obvious differences between rhizosphere sediment and sediment bacterial communities. The result of nMDS also appeared to be two clearly differentiated plates (Figure 1C).

The result of nMDS (constrained by treatment) and ANOSIM highlighted the biochar effect on all bacteria communities (Figure 1C). Pairwise tests revealed significant differences (*p*<0.05) between SB and SC, while there were no significant differences between RSB and RSC. Meanwhile, it also revealed that the community composition changed significantly with time.

### Relative Abundance of the Different Classification Level

In general, most bacteria were gram-negative (91.49%±0.29 and 85.97%±1.16% for rhizosphere and sediment bacterial communities, respectively; Figure 1B). Phylum Bacteroidetes (6.74%±0.96 and 11.03%±1.37% for rhizosphere and sediment bacterial communities, respectively) and Proteobacteria (73.54%±3.11 and 67.26%±1.48% for rhizosphere sediment and sediment bacterial communities, respectively) were the most two abundant phyla for all the samples (Figure 1B). Phylum Cyanobacteria was much more abundant in the rhizosphere sediment (6.07%±2.22%) than sediment (2.25%±1.16%).

**Figure 2:**
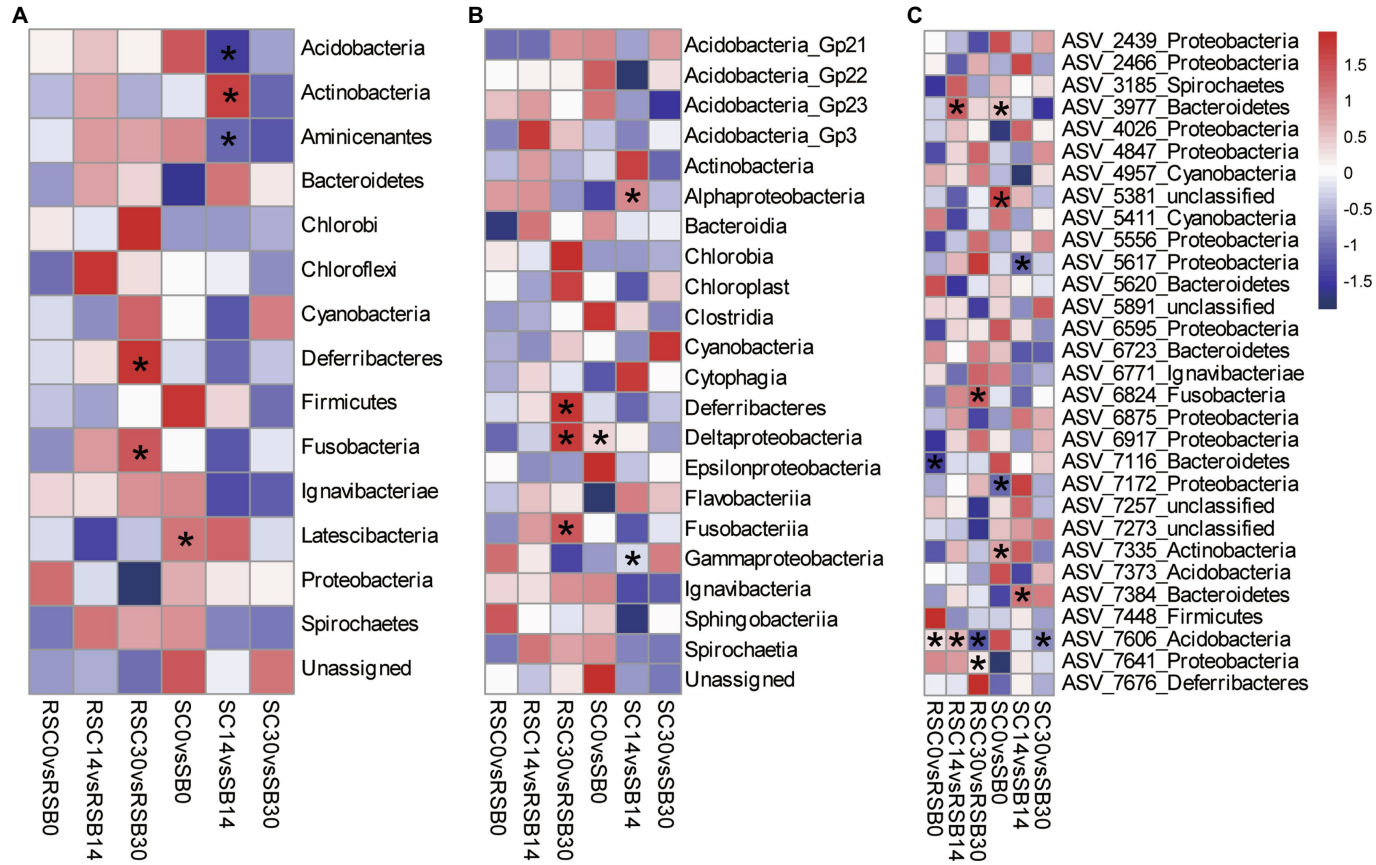
Heatmap of differences between different groups at Phylum **(A)**, Class **(B)** and ASV (**C**: 30 ASVs with the highest relative abundance) level, respectively. The value represented by the color was calculated by Welch’s *t* test in STAMP, value >0 (red) means control group has a higher abundance, while value <0 (blue) means biochar addition group has a higher abundance. (^*^p < 0.05).

At the phylum level, Phylum Deferribacteres and Fusobacteria decreased significantly with biochar addition for rhizosphere sediment bacterial communities on day 30. While for sediment bacterial communities, Phylum Actinobacteria decreased, while Acidobacteria and Aminicenantes increased significantly with biochar addition on day 14 (Figure 2A).

Moreover, Class Deferribacteres, Deltaproteobacteria, and Fusobacteriia decreased significantly with biochar addition for rhizosphere sediment bacterial communities on day 30. Compared with the control group, the relative abundance of Class Alphaproteobacteria with biochar addition group was lower, while Class Gammaproteobacteria were higher on day 14 for sediment bacterial communities (Figure 2B).

At the ASV level, ASV7606 affiliated with the Phylum Actinobacteria showed significant differences between the control and biochar addition groups for both rhizosphere sediment and sediment bacterial communities. In addition, ASV7641 (Phylum Proteobacteria), ASV6824 (Phylum Fusobacteria), and ASV3977 (Phylum Bacteroidetes) presented significant differences between groups for rhizosphere sediment bacterial communities. In contrast, ASV7384 (Phylum Bacteroidetes) and ASV5617 (Phylum Proteobacteria) exhibited differences for sediment bacterial communities (Figure 2C).

### Relative Abundances of Genes Involved in the Nutrient Cycle

The relative abundances of genes involved in carbon fixation, phosphorus and sulfur metabolism, and nitrogen cycle were predicted by PICRUSt2 (Supplementary Figure S5A). The abundance of some function genes (e.g., *acsB* for carbon fixation; *ugpQ*, *glpA*, *glpD,* and *glpK* for phosphorus metabolism; *sat*_*met3* and *cysH* for sulfur metabolism; *nifD*, *nirK,* and *nosZ* for nitrogen cycle) was significantly different between the rhizosphere sediment and the sediment bacterial communities (Supplementary Figure S5B).

**Figure 3:**
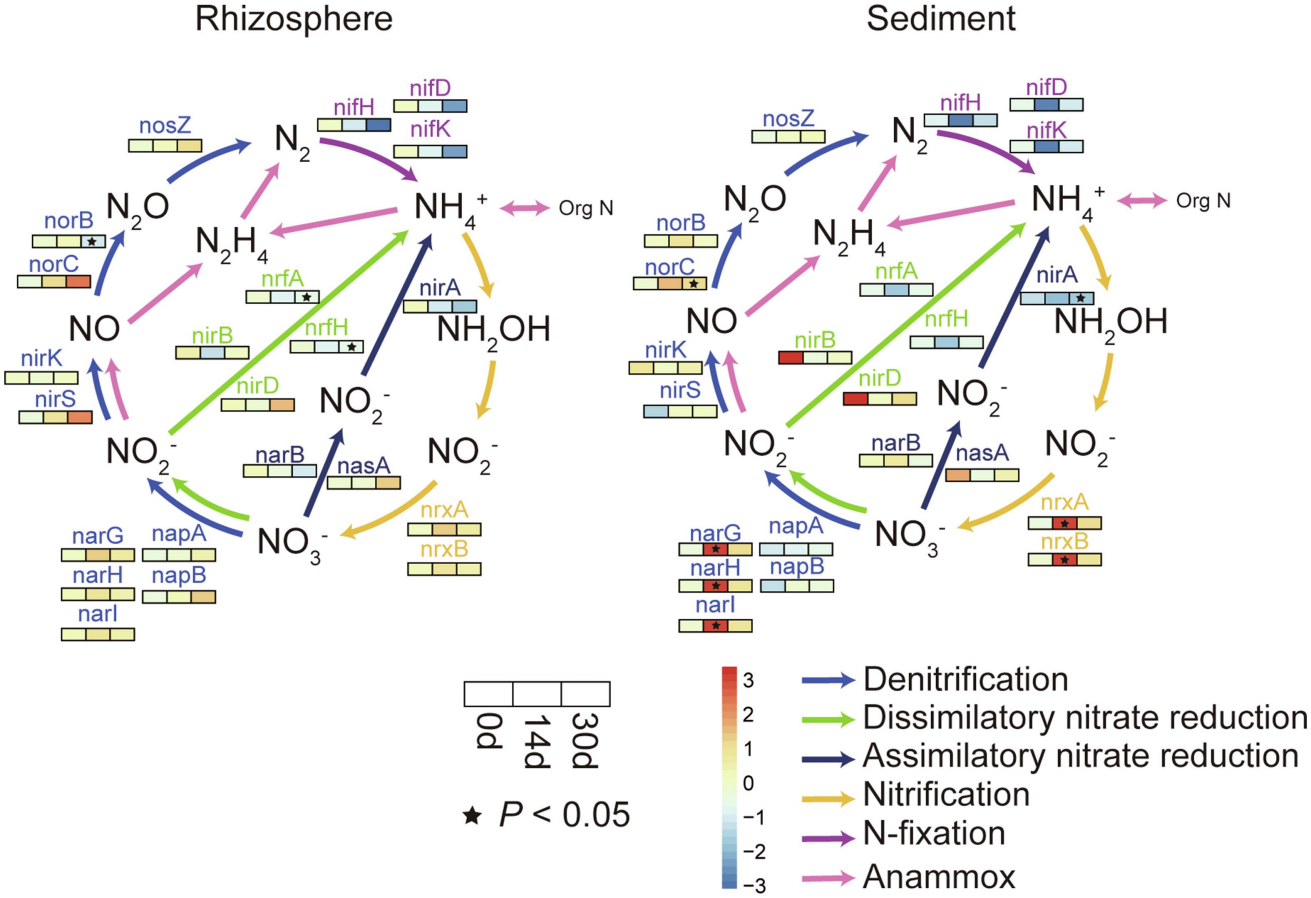
Relative changes of N-cycling genes in rhizosphere and sediment. For each subfigure, the value represented by the color was calculated by Welch’s *t* test in STAMP, colors indicate relative differences in gene abundance between the biochar addition groups and control groups, value >0 (red) means biochar addition group has a higher abundance, while value <0 (blue) means control group has a higher abundance. (^*^p<0.05).

As illustrated in Supplementary Figure S6, both the rhizosphere sediment and sediment bacterial communities showed a significant positive correlation (*p*<0.05) with NO_3_-N. Therefore, in order to better identify the effect of biochar on nitrogen cycling genes, relative changes of nitrogen cycling genes between control and biochar addition groups were calculated in Figure 3. Effects of biochar addition on nitrogen cycling genes of the rhizosphere sediment and sediment were different. In sediment, these genes appeared significantly different on day 14 principally. For instance, the nitrogen fixation genes (*nifH*, *nifD,* and *nifK*) were restrained by biochar addition, and denitrification genes (*narG*, *narH,* and *narI*) and nitrification genes (*nrxA* and *nrxB*) were significant increased on day14. In the rhizosphere sediment, the biochar exerted a primary effect on day 30, and the abundance of nitrogen fixation genes (*nifH*, *nifD*, and *nifK*) was decreased. Besides, biochar addition also significant decreased the abundance of dissimilatory nitrate reduction genes (*nrfA* and *nrfH*).

### Ecological Processes Influencing Bacterial Community Assembly

**Figure 4:**
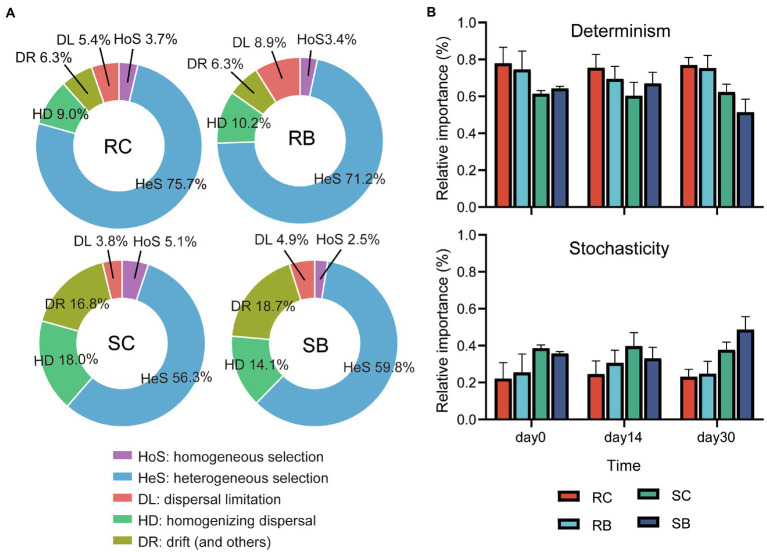
Relative importance of different ecological processes in response to biochar addition. **(A)** Community assembly processes of bacterial community from different groups. **(B)** Changes of determinism and stochasticity; Data are presented as mean values±SD. Error bars represented standard deviations; For RC, *n*=6 comparisons among four biologically independent samples at each time point; For SC, RB and SB, *n*=3 comparisons among three biologically independent samples at each time point. [Determinism: HoS+HeS; stochasticity: DL+HD+DR (Ning et al., 2020)].

It has been proved that both niche and habitat filtering have effects on bacterial community structure. Null model analysis was used to disentangle the relative importance of stochastic and deterministic processes (homogenous and heterogeneous selections) in the microbial assembly within biochar addition sediment of seagrass (Stegen et al., 2013). The determinism process showed a stronger impact on the community assembly for bacteria than stochasticity in all groups, and heterogeneous selection dominated the deterministic process (Figure 4A). Compared to sediment bacterial communities, the determinism process showed a stronger impact on rhizosphere bacterial communities. Biochar addition led to a higher relative importance of stochasticity in sediment on day 30, while there was barely any effect on rhizosphere bacterial communities (Figure 4B).

### Co-occurrence Patterns of the Bacterial Communities

**Figure 5:**
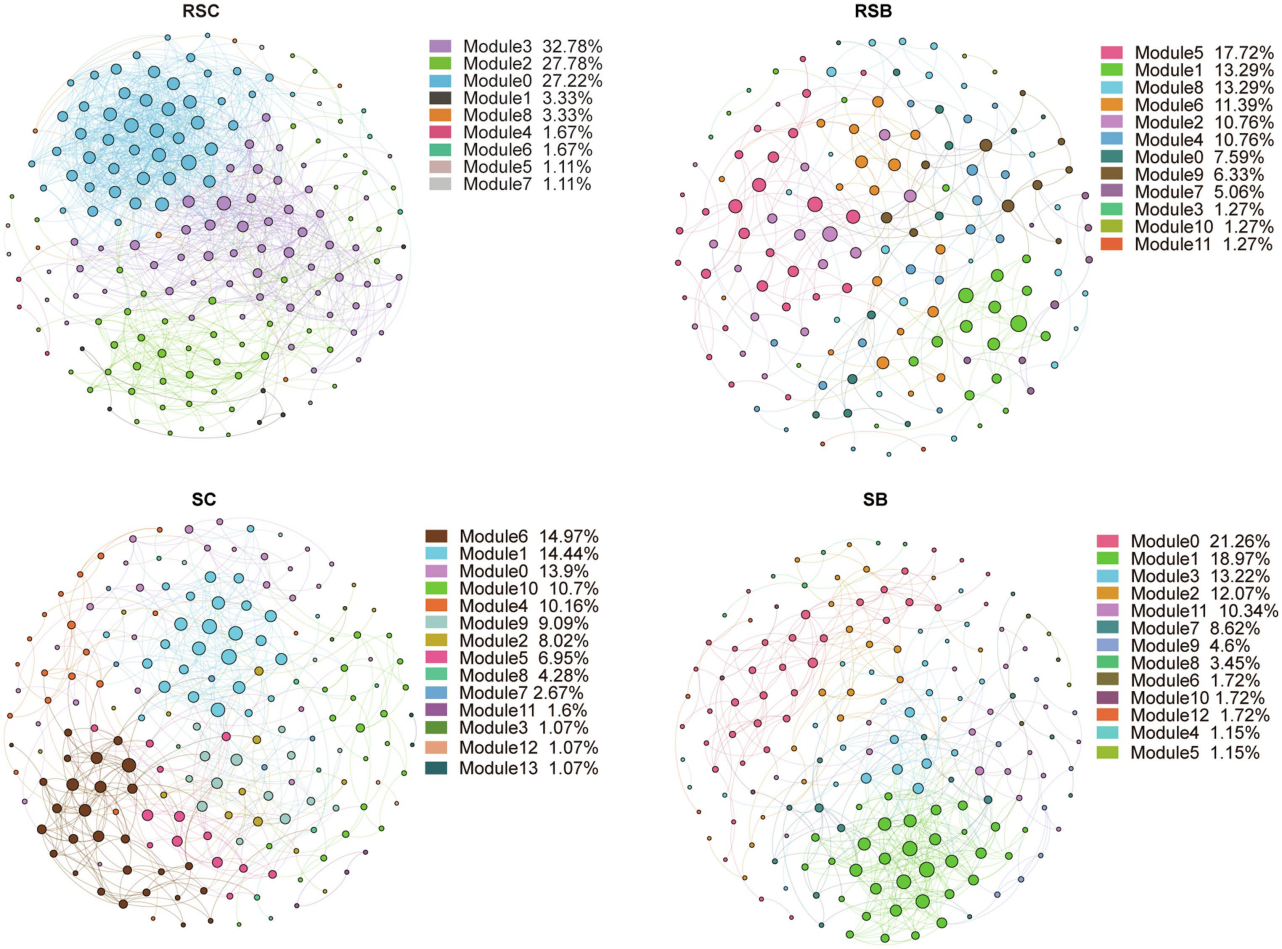
Species-species and species-environment association network. A connection stands for a strong (Spearman’s |*r*|>0.6) and significant (value of *p*<0.01) correlation.

The bacterial community composition co-occurrence networks were constructed to identify the ecological interplay between co-occurrence taxa (Figure 5). Network topological features showed that the co-occurrence pattern in the rhizosphere sediment differed from the sediment network. There was a substantial change in the rhizosphere sediment bacterial community network with biochar addition, while the network topological features showed a minor fluctuation. After filtering most ASVs of low abundance, the final network had 180, 158, 187, and 174 nodes for RSC, RSB, SC, and SB, respectively. Network density, clustering coefficient, and average numbers of degrees were lower, while module numbers were higher for RSB than RSC (Supplementary Table S3).

The modular structure of the co-occurrence network was compared between RSC, RSB, SC, and SB. The RSC, RSB, SC, and SB networks parsed into three, six, five, and five major modules (modules with nodes number more than 10% of total nodes number), respectively, which accounted for 87.8, 77.2, 64.2, and 75.9% of their corresponding networks. The modules of RSC and RSB were primarily occupied by Class Deltaproteobacteria and Gammaproteobacteria, while the modules of SC and SB were primarily occupied by Class Deltaproteobacteria, Gammaproteobacteria, and Bacteroidetes (Supplementary Figure S7).

Topologically, the nodes represent distinct roles in the network. Ecologically, module hubs and connectors signify generalists, network hubs indicate supergeneralists, while peripherals represent specialists. Keystone taxa were identified and displayed *via* Zi-Pi plots (Supplementary Figure S8). There were no module hubs and network hubs for all networks (Supplementary Figure S8). About 16.7, 8.9, 16.6, and 14.4% of ASVs in the RSC, RSB, SC, and SB were connectors, respectively, and most of them were affiliated with the Phylum Proteobacteria (Supplementary Table S4).

## Discussion

### Biochar Addition Changed the Environment and Improved the Photosynthetic Rate of Seagrass

Biochar addition changed some environment variables significantly in this study. There was a significant pH increase with biochar addition. Over time, the pH of biochar in the sediment may change and either decrease or increase depending on the biochar type. Nguyen and Lehmann (2009) observed a pH decrease with mineral-poor oak wood biochar from pH 4.9 to 4.7, but an increase with mineral-rich corn stover biochar from pH 6.7 to 8.1 during one-year incubation. The driving force behind a pH decrease is the oxidation of carbon to form acidic carboxyl groups (Cheng et al., 2006), whereas the increase in pH is likely related to the dissolution of alkaline minerals. Elevated pH caused by biochar addition might benefit bacteria over fungi (Rousk et al., 2009).

Biochar can affect the microbially-mediated transformation of nutrients significantly in the soil, and it could increase the adsorption of NO_3_-N (Van Zwieten et al., 2010) and the soil contents of NO_3_-N and TN (Li et al., 2020b). However, there was a minor decrease for NO_3_-N while a significant decrease for TN with biochar addition in this study. In agricultural systems, the higher concentrations of available kalium would likely encourage plant uptake of NO_3_-N (Chan et al., 2007), which led to a lower concentration of NO_3_-N in the sediment of biochar addition groups. Van Zwieten et al. (2010) also found that biochar addition could increase kalium, which was consistent with our results.

Supplementary Figure S1 presents that the phosphorus in the sediment significantly increased in the biochar addition group. Moreover, Lu et al. (2020) found that the biochar addition increased the abundance of genes involved in inorganic phosphate solubilization and organic phosphorus mineralization, but not those involved in phosphorus transportation of the phosphorus cycling. The microbial activities related to organic phosphorus mineralization were enhanced by biochar addition (Masto et al., 2013). Besides, biochar addition at a rate of 20gkg^−1^ soil increased acid phosphatase activity (+32%) and alkaline phosphatase activity (+22.8%; Masto et al., 2013).

On day 30, there was a significant increase in the biochar addition group’s *in situ* seagrass photosynthetic performance, of which a higher value means a strong photosynthetic performance. Higher photosynthetic performance could enhance seagrass growth and optimize CO_2_ utilization. This result was consistent with our hypothesis that biochar addition would improve the health of the seagrass.

### Biochar Addition Changed the Rhizosphere Sediment Bacterial Community Composition With Delayed Effect, but Not Alpha Diversity

Biochar addition could significantly change the bacterial community compositions (Lehmann et al., 2011; Xu et al., 2014; Zhang et al., 2018; Wei et al., 2020). The specific sets of microbes in the rhizosphere and sediment demonstrated clear separation by compartment in the nMDS plot. This distinctiveness of the plant rhizosphere microbiome was also found in previous studies (Bulgarelli et al., 2012; Lundberg et al., 2012; Peiffer et al., 2013; Yeoh et al., 2015; Zarraonaindia et al., 2015; Hartman et al., 2017). Plants recruit a rhizosphere sediment microbiome in their early life stages from a larger pool of sediment microbes. The initial composition of this sediment microbial pool is the most influential factor determining the composition of rhizosphere sediment microbial communities (Hartman et al., 2018). Therefore, the investigation on the response of both rhizosphere sediment microbiome and sediment microbes to the biochar addition was necessary. In this study, biochar exerted distinct effects on sediment bacterial communities than rhizosphere sediment bacterial communities. The differences between the control and biochar addition groups appeared on day 30 for rhizosphere sediment bacterial communities and day 14 for sediment bacterial communities. Taken together, biochar may have a direct and rapid effect on the sediment bacterial communities, and the change of sediment bacterial pool led to rhizosphere sediment bacterial communities change with a “delayed effect.”

Biochar addition could increase the sediment bacterial alpha diversity in constructed wetlands (Deng et al., 2019) and improve microbial activity in PAH-stressed soil (Li et al., 2020b). However, there were no significant differences in alpha diversity between control and biochar addition groups in this study. This may be explained by the different environments and different biochar properties. Effects of biochar application on soil bacterial community structure variations and activities remain controversial under different biochar characteristics, soil properties, and experiment conditions. The role of biochar in soil biological processes, therefore, represents a frontier in soil science research with many unexplained phenomena awaiting exploration.

### Three Patterns of Bacterial Groups Induced by Biochar Addition

Phylum Bacteroidetes and Proteobacteria were the two most abundant phyla detected in this study. Their dominance was also observed in previous large-scale surveys of soil microorganisms (Fierer et al., 2007; Lauber et al., 2009). While this study reported the general characteristics of bacterial communities, it also revealed some specific patterns. For example, biochar addition groups with the oligotrophic environment had a higher relative abundance of Acidobacteria in sediment on day 14, which preferred oligotrophic soils (Fierer et al., 2007), and they did not seem to have outcompeted in soils of high CO_2_ concentration plots despite with the increased flux of C (Austin et al., 2009).

In the present study, there were three changing patterns of all subgroups based on three sampling time points: Pattern 1: Rhizosphere sediment and sediment bacterial subgroups had consistent variation trend; Pattern 2: rhizosphere sediment and sediment bacteria subgroups had reverse variation trend; and Pattern 3: no correlation between changes of rhizosphere sediment and sediment bacteria subgroups (Supplementary Figure S9).

Pattern 1: The biochar addition may create a similar environment for some subgroups, and the relative abundance of these subgroups will increase or decrease depending on the environment and their competitor in the same niche with a similar trend in rhizosphere sediment and sediment. For example, biochar addition caused the relative abundance of phylum Actinobacteria to decrease on day 14 while increasing on day 30, and that of phylum Cyanobacteria increased on day 14 while decreasing on day 30 in both rhizosphere and sediment.

Pattern 2: The biochar addition may create an uneven environment and have different effects on the sediment and rhizosphere sediment. Subsequently, some bacterial groups moved from the rhizosphere sediment to the sediment, while some bacterial groups moved from the sediment to the rhizosphere sediment driven by environmental factors. For example, biochar addition caused a decrease in the relative abundance of Phylum Fusobacteria and Aminicenantes in the rhizosphere sediment while an increase in sediment on day 30. Fusobacteria is a phylum of obligately anaerobic bacteria commonly found in marine sediment environments (Hofstad et al., 1991), and they have putative hydrocarbon-degrading qualities (Gutierrez et al., 2016). On the other hand, Phylum Aminicenantes exhibited the highest relative abundance in hydrocarbon-impacted environments, followed by marine habitats (especially hydrothermal vents and coral-associated microbiome samples; Farag et al., 2014). Taken together, a possible reason for this might be that biochar addition increased hydrocarbon concentration in the sediment or decreased hydrocarbon concentration in the rhizosphere sediment, which led to some hydrocarbon-related bacteria moving away from the rhizosphere.

Pattern 3: These subgroups may be governed by pattern 1 and pattern 2 with a combined effect, making their variation trends irregular. For example, Phylum Proteobacteria showed a similar variation trend before day 14, while had a reverse trend on day 30. In this study, only three sampling times were set. To further investigate the variation trends of different subgroups, more time points needed to be set.

### Biochar Changed Nutrient Cycles, Especially the Nitrogen Cycle

Biochar addition significantly changed some bacterial groups involved in nutrient cycling. For example, the relative abundance of Phylum Deferribacteres decreased significantly with biochar addition in rhizosphere sediment bacterial communities on day 30. The previous study showed that Phylum Deferribacteres might have the *nifH* gene, which is important for nitrogen fixation (Zehr et al., 2003). Moreover, the relative abundance of Phylum Actinobacteria decreased significantly with biochar addition on day 14 for sediment bacterial communities. In the rhizosphere sediment, the enrichment of Actinobacteria could improve bacterial activity and nutrient cycling (Koranda et al., 2011; Zhang et al., 2019). Compared with the control group, the relative abundance of Class Alphaproteobacteria was lower on day 14 for sediment bacterial communities in the treatment group. Bacteria of Class Alphaproteobacteria frequently adopted an intracellular lifestyle as plant mutualists or plant or animal pathogens (Batut et al., 2004). A variety of metabolic strategies are found in this class, including photosynthesis, nitrogen fixation, ammonia oxidation, and methylotrophy (Williams et al., 2007).

The microbiota, mainly bacteria and archaea, drives the soil nitrogen cycle. Many investigations have been carried out on the effects of biochar application on soil microbiota (Kolton et al., 2011; Chen et al., 2012), and biochar addition improved the nitrogen cycle by changing the bacteria community composition (Chan et al., 2007, 2008; Major et al., 2009). Biochar addition restrained nitrogen fixation genes while promoting the transform between ${\mathrm{NO}}_{3}^{-}$ and ${\mathrm{NO}}_{2}^{-}$ in this study. Furthermore, Xiao et al. (2019) found that biochar addition significantly increased the abundance of ammonia-oxidizing archaea (AOA), *nirK*, *nirS,* and *nosZ* by an average of 25.3, 32.0, 14.6, and 17.0%, respectively. Biochar addition may improve both nitrification and denitrification and accelerate nitrogen cycling. The increased activity of nitrifying microorganisms in biochar may be due to the increase of ammonium nitrogen and DOC contents (Mierzwa-Hersztek et al., 2018). At the same time, DOC drives the turnover of C and N in microorganisms, which stimulates the growth of microorganisms and promotes the activity of denitrifying enzymes (Xiao et al., 2019). Furthermore, nitrification is an acidifying process (Bolan et al., 1991). The alkaline biochar may create much more favorable conditions for nitrifiers and then increase nitrification rates due to its liming effect (Prommer et al., 2014; Ulyett et al., 2014). Nishio (1996) and Rondon et al. (2007) found biochar addition to soil increased biological nitrogen fixation. In addition, Wu et al. (2020) found biochar decreased the diversity of the diazotrophic community and altered diazotroph community structure during composting. Biochar changed the community structure of nitrogen-fixing bacteria, but the effect on *nifH* gene abundance was not clearly determined.

### NO_3_-N but Not pH as the Key Driver of Both Rhizosphere Sediment and Sediment Bacteria Communities

Recent studies suggested that the richness and diversity of the soil bacterial communities were strongly related to soil pH (Nicol et al., 2008; Lauber et al., 2009; Li et al., 2020b). However, there was no significant correlation between the bacterial community’s taxonomical composition and pH. The “size-plasticity” hypothesis argues that smaller individuals are less environment filtered than larger individuals because smaller individuals are more likely to have plasticity in metabolic abilities (Finlay, 2002; Langenheder et al., 2005). Therefore, bacteria may exist widely in such a narrow pH range, suggesting that the selection pressure of pH was invisible on the bacterial community.

In our result, both the rhizosphere sediment and sediment bacterial communities showed a significant positive correlation (*p*<0.05) with NO_3_-N concentration. One possible explanation was that biochar directly affected NO_3_-N concentration (Sui et al., 2021); then, NO_3_-N acted on bacterial communities. Another explanation was that biochar could act on nitrogen-related bacteria and seagrass (Lehmann et al., 2011; Yu et al., 2021), and the variations in bacteria and seagrass changed the concentration of NO_3_-N afterward.

### Biochar as a Stabilizer to the Original Environment

In this study, the distinct community assembly pattern of bacterial communities could be mainly explained by deterministic processes rather than stochastic processes, which supported the result found by Logares et al. (2013) that deterministic processes dominated the biogeography of bacterial communities which exposed to progressive long-term environmental change in coastal lakes. Previous studies also showed bacteria were predominantly structured by selection, while microeukaryotes were mainly structured by drift (Logares et al., 2018; Mo et al., 2020).

The biochar addition barely had any effect on the community assembly pattern of seagrass rhizosphere sediment bacterial communities, and it even led to a decreased importance of deterministic processes in sediment bacterial communities. Logares et al. (2018) found three phases of a community that changed after a disturbance. Phase 1: Stochastic processes initially governed by microbial community assembly. Phase 2: Changes in the local environment progressively increased the importance of deterministic selection. Phase 3: The emergence of stable environments led to stable levels of deterministic selection. Selection derived from the variations in the reproductive success across individuals and species caused by the biotic and abiotic pressures; the constant and reduced importance of deterministic selection meant biochar addition might act as a stabilizer to the original environment.

### Biochar Addition Changed the Bacterial Co-occurrence Pattern

From the perspective of biotic factors, the relationships between microorganisms exert considerable influence and are also an important aspect of selection pressures. Network structure has important implications for the co-occurrence of species and their stability (Bascompte et al., 2003). The RSC network structure was more complex than RSB with more nodes and edges, while there were slight differences between SC and SB (Figure 5, Supplementary Table S3). In general, a more complex network structure may indicate more stable co-existence patterns, and a stable co-occurrence pattern mirrored fewer dynamic characteristics to some extent (Costa et al., 2006). Thébault and Fontaine (2010) demonstrated that high connectivity promoted community stability in mutualistic networks. SB had a lower network density and clustering coefficient with higher module numbers, which meant that the community was separated into more independent groups.

There were several keystones in our network, and all of them were connectors. The loss of these species may lead to the breakdown of the ecological networks and modules (Guimerà and Amaral, 2005). Therefore, these potential key species might be crucial in maintaining the stability of the bacterial communities. The identified connector taxa in the RSC/RSB and SC/SB were quite different, but they were primarily from Phylum Proteobacteria. Hence, they may have similar ecosystem functions.

However, these modules from all networks did not necessarily reflect their taxonomic classification. Most bacterial interactions were stronger between phyla/classes than within phylum/class, which provided evidence that the bacterial community structure is shaped by environmentally driven functional characteristics rather than phylogeny (Burke et al., 2011).

## Conclusion

Our study investigated for the first time the influence of biochar addition on the bacterial relative abundance, composition, assembly, and co-occurrence network of bacterial communities in the seagrass ecosystem. Rhizosphere sediment and sediment bacterial communities responded differently to the biochar addition. The significant bacterial community composition changes in rhizosphere sediment occurred after incubation for 30days with a delay effect than that of in sediment (14days). Alteration of environmental factors and biotic interactions induced by biochar addition enhanced nitrification and denitrification, which may accelerate nitrogen cycling. More nitrogen absorption and photosynthetic performance of seagrass after biochar addition may lower the total nitrogen in sediment. Together, biochar addition could improve seagrass health, which has important implications for biochar application in the seagrass ecosystem.

## Data Availability Statement

The data sets presented in this study can be found in online repositories. The names of the repository/repositories and accession number(s) can be found online at: https://www.ncbi.nlm.nih.gov/, PRJNA750881.

## Author Contributions

JL and JD: conceptualization, methodology, resources, and supervision. WZho: investigation. JZ and JL: formal analysis and data curation. JZ: writing – original draft and visualization. All authors: writing – review and editing.

## Funding

The research was supported by the National Natural Science Foundation of China (41676163), Key Special Project for Introduced Talents Team of Southern Marine Science and Engineering Guangdong Laboratory (Guangzhou; GML2019ZD0402), National Key Research and Development Program of China (2018YFC1406505, 2018FY100105, and 2017YFC0506301), Innovation Academy of South China Sea Ecology and Environmental Engineering, Chinese Academy of Sciences (ISEE2021ZD03), and Science and Technology Planning Project of Guangdong Province, China (2020B1212060058).

## Conflict of Interest

The authors declare that the research was conducted in the absence of any commercial or financial relationships that could be construed as a potential conflict of interest.

## Publisher’s Note

All claims expressed in this article are solely those of the authors and do not necessarily represent those of their affiliated organizations, or those of the publisher, the editors and the reviewers. Any product that may be evaluated in this article, or claim that may be made by its manufacturer, is not guaranteed or endorsed by the publisher.

## Footnotes

^1^ https://github.com/taowenmicro/ggClusterNet

^2^ https://github.com/DaliangNing/iCAMP1
