# Supplementary material for: Biochar Addition Altered Bacterial Community and Improved Photosynthetic Rate of Seagrass: A Mesocosm Study of Seagrass Thalassia hemprichii
Source: Front Microbiol. 2021 Dec 2;12:783334. doi: 10.3389/fmicb.2021.783334 (PMC8678274; doi:10.3389/fmicb.2021.783334)
Supplement: Supplementary file 1 [file Data_Sheet_1.docx]

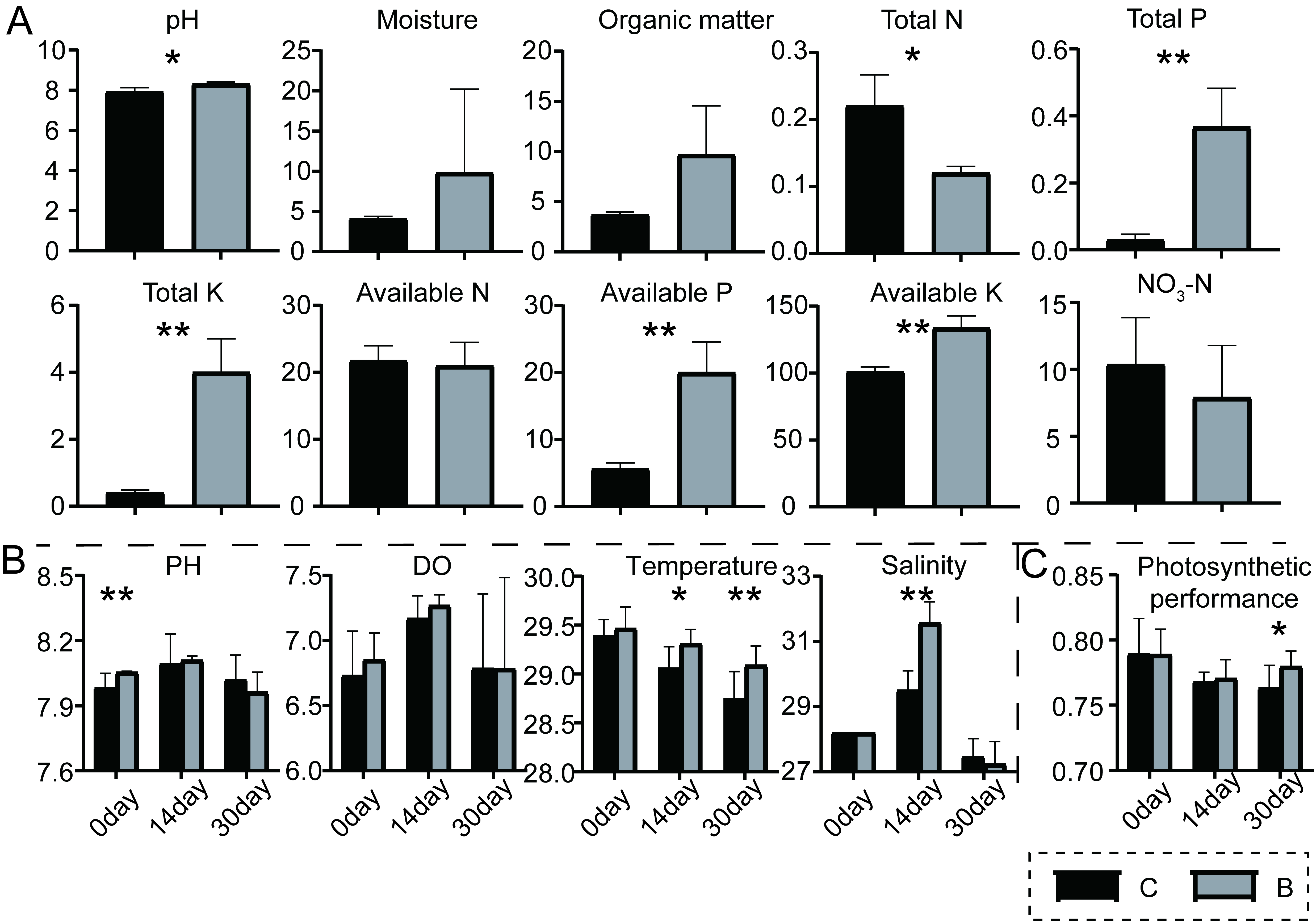


**Fig. S1.** Some physiochemical variables were detected in the experiment. (C: control groups; B: biochar addition groups).

A. Environmental variables of sediment on day 30 (N: nitrogen; P: phosphorus; K: kalium).

B. Environmental variables of water on day 0, day 14 and day 30.

C: Photosynthetic performance of seagrass on day 0, day 14 and day 30.


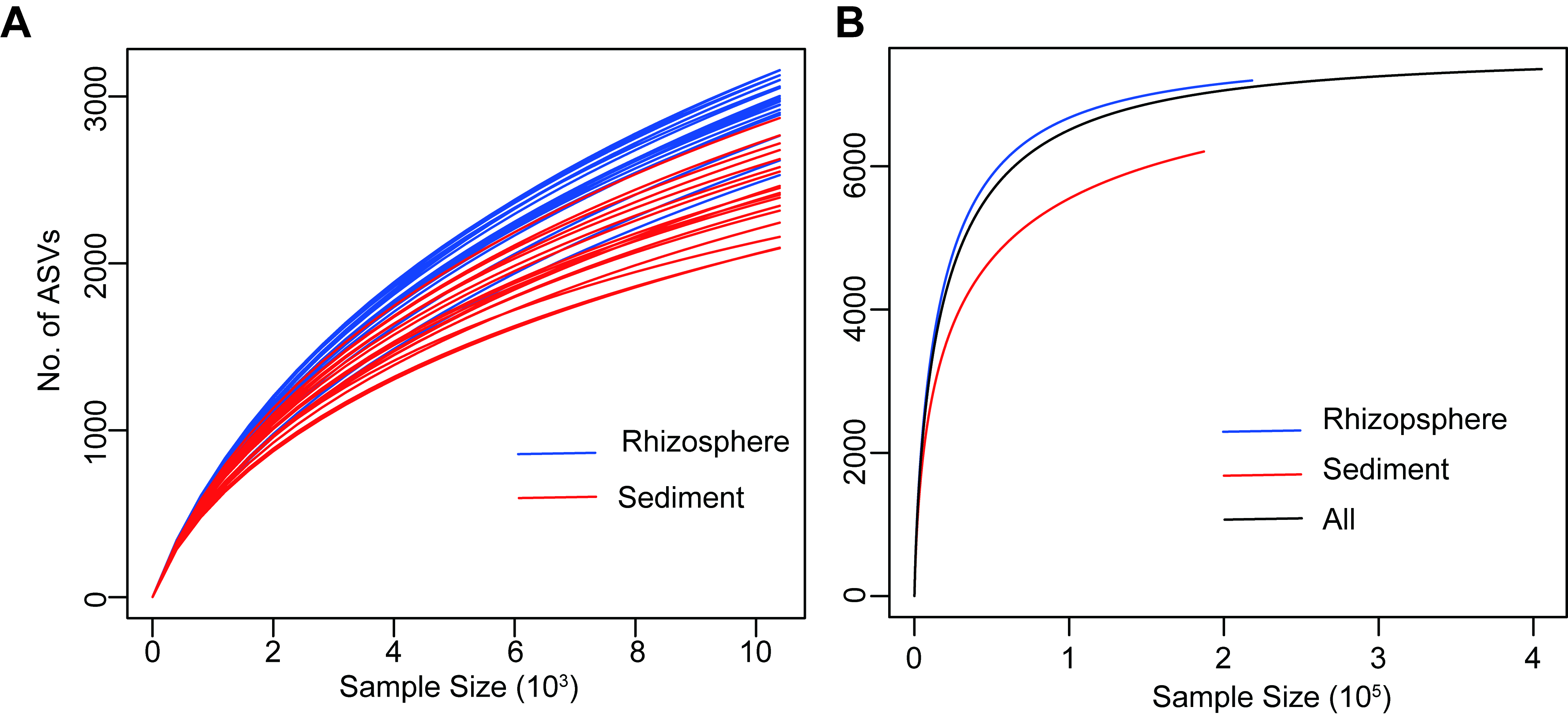


**Fig. S2.** Rarefaction curves of similarity-based amplicon sequence variants (ASVs) at 100% sequence identity threshold.

A. The individual bacterial samples.

B. The combined sets of rhizosphere and sediment bacterial samples.


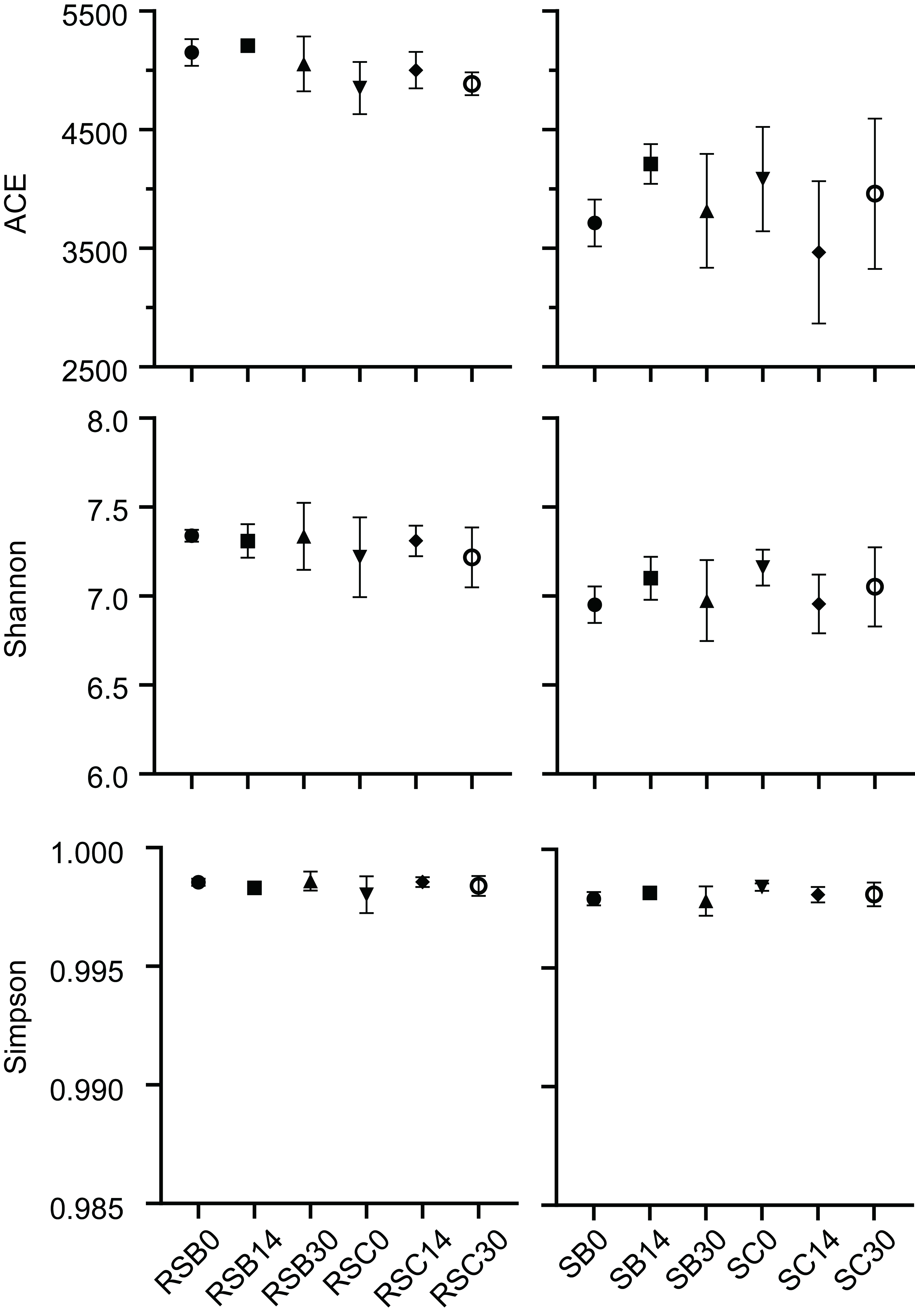


**Fig. S3.** Bacterial alpha diversity of different subgroups (there was no significant difference between groups with *P* > 0.050).


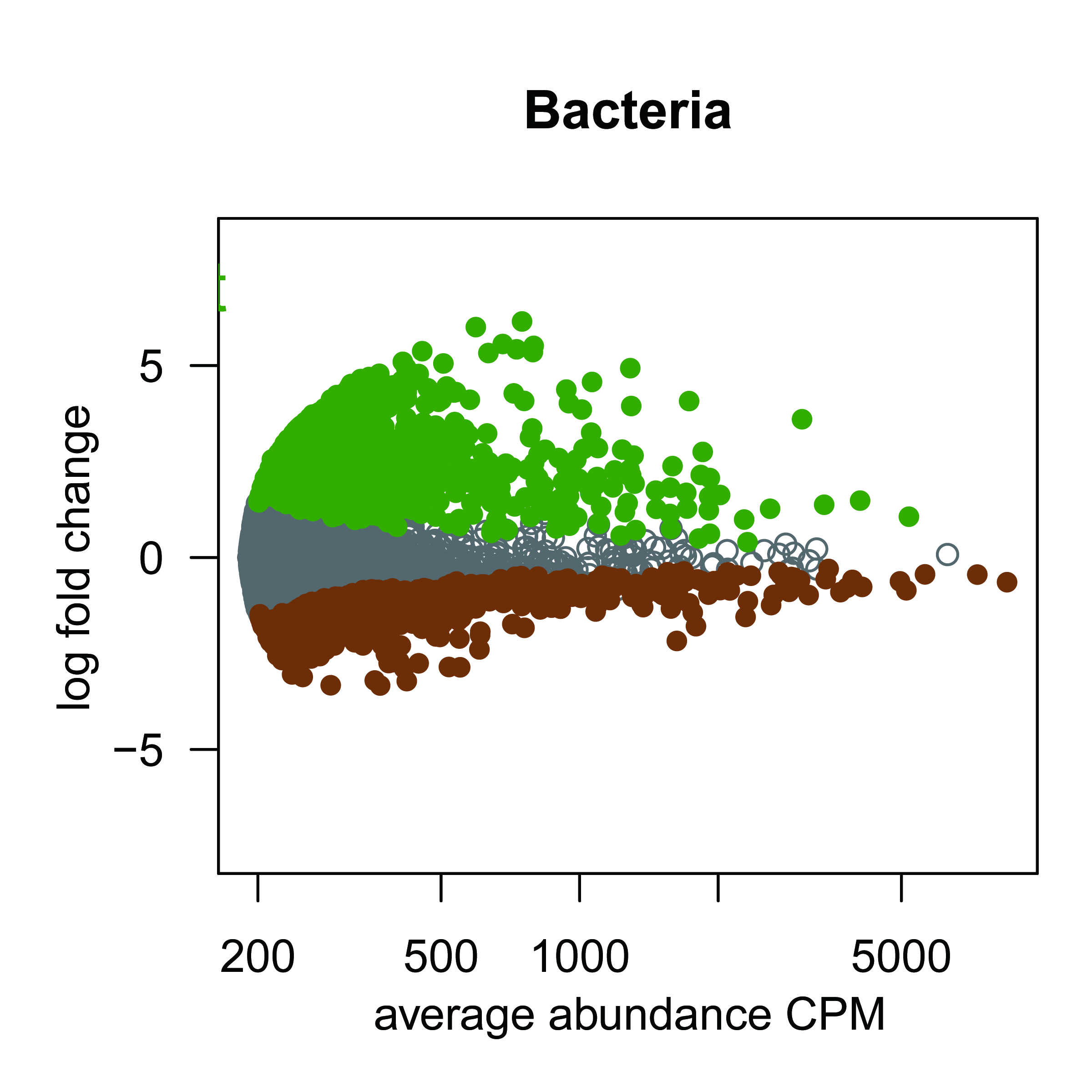


**Fig. S4.** Sediment and roots harbor specific sets of microbes. MA plots displaying the abundance patterns of bacteria and fungi in soil and root microbiomes. X-axis reports average OTU abundance (as counts per million, CPM), and Y-axis log2-fold change (root relative to soil). Root and soil-specific OTUs were colored in green and brown, respectively, and non-differentially abundant OTUs are in gray (likelihood ratio test, p < 0.05, FDR corrected).


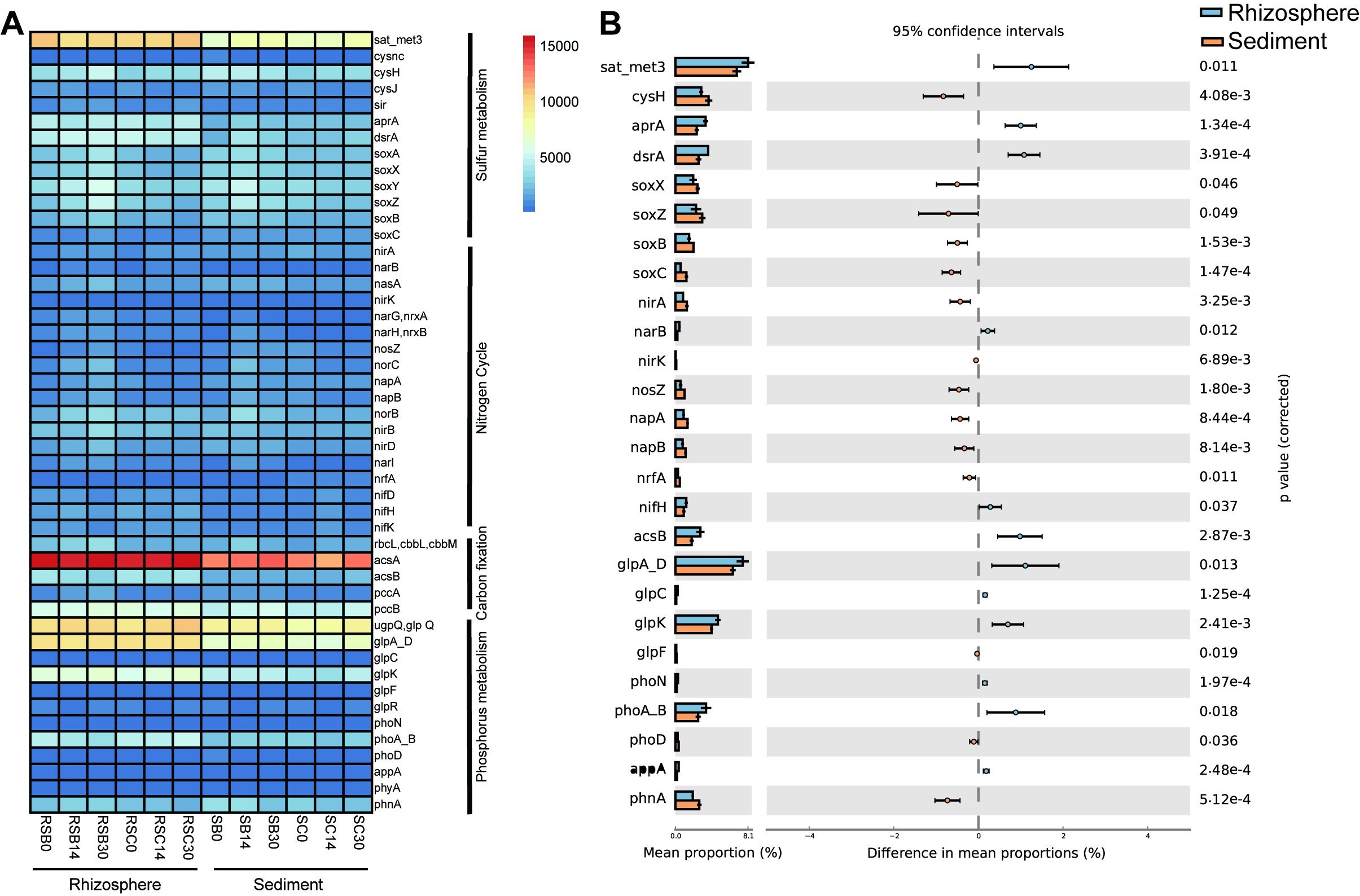


**Fig. S5.** Abundance and Welch’s t-test in STAMP of 13 sulfur metabolism genes, 18 nitrogen cycle genes, 5 carbon fixation genes and 12 phosphorus metabolism genes

A. Heatmap of gene abundance in different group of samples.

B. Genes which were significant different between rhizosphere and sediment (*P* < 0.05).





**Fig. S6.** Correlation between different environmental variables and correlation between environmental variables and bacterial communities (Only significant correlation were presented).


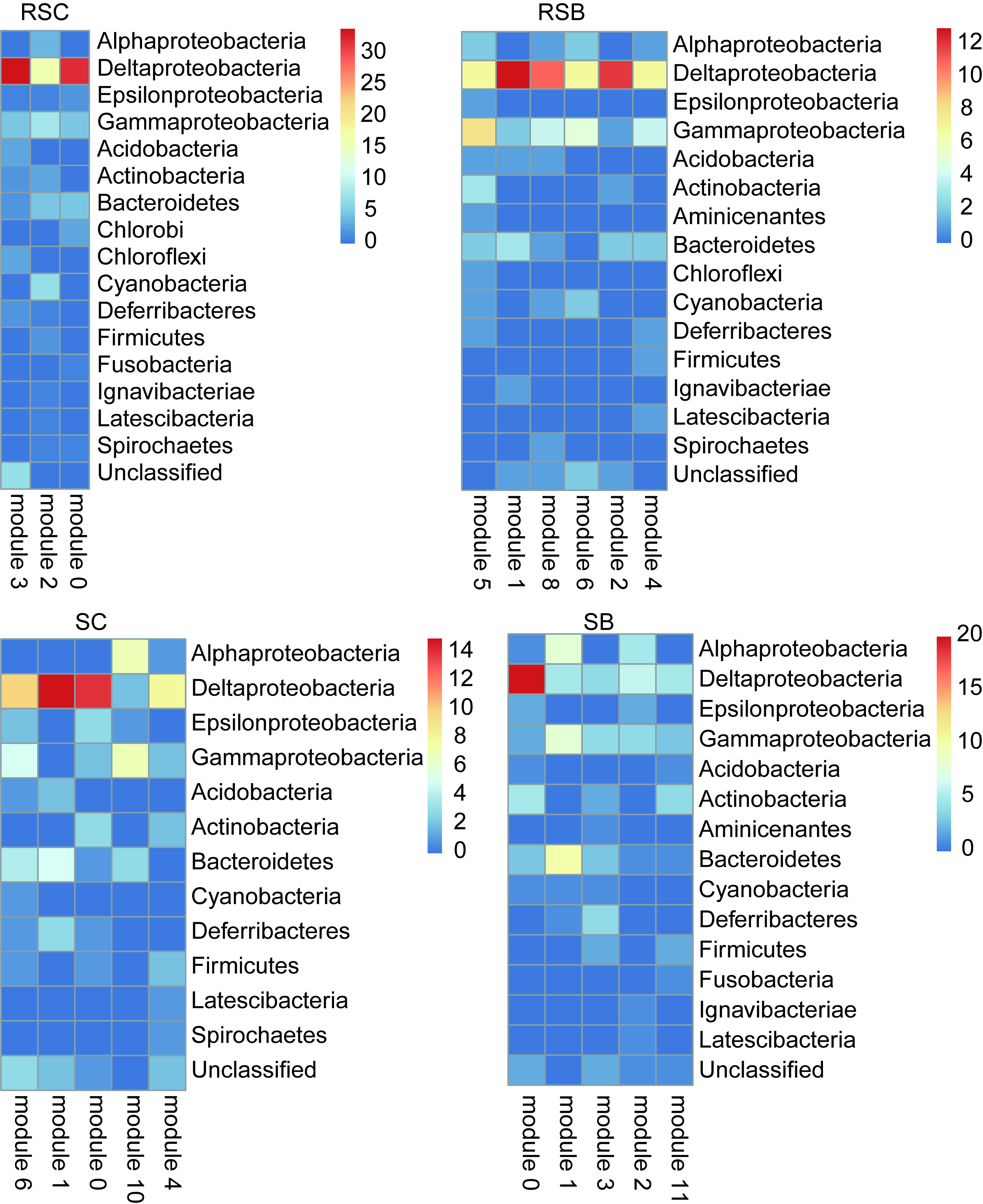


**Fig. S7.** Heatmap showing the relative abundance of the subgroups (Phylum and Class level) within each module for every networks.


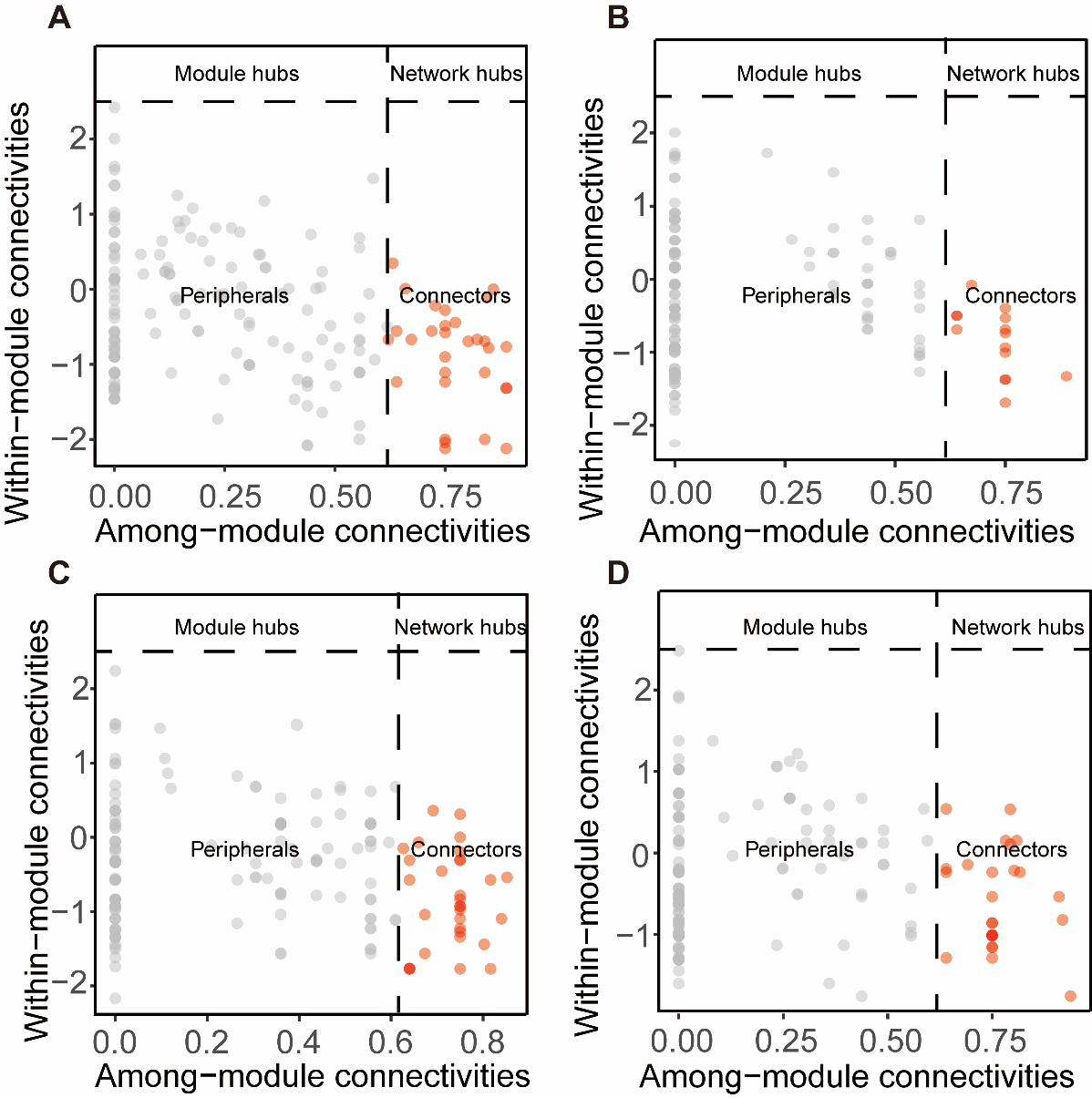


**Fig. S8.** Zi‐Pi plot showing the distribution of ASVs based on their topological roles.


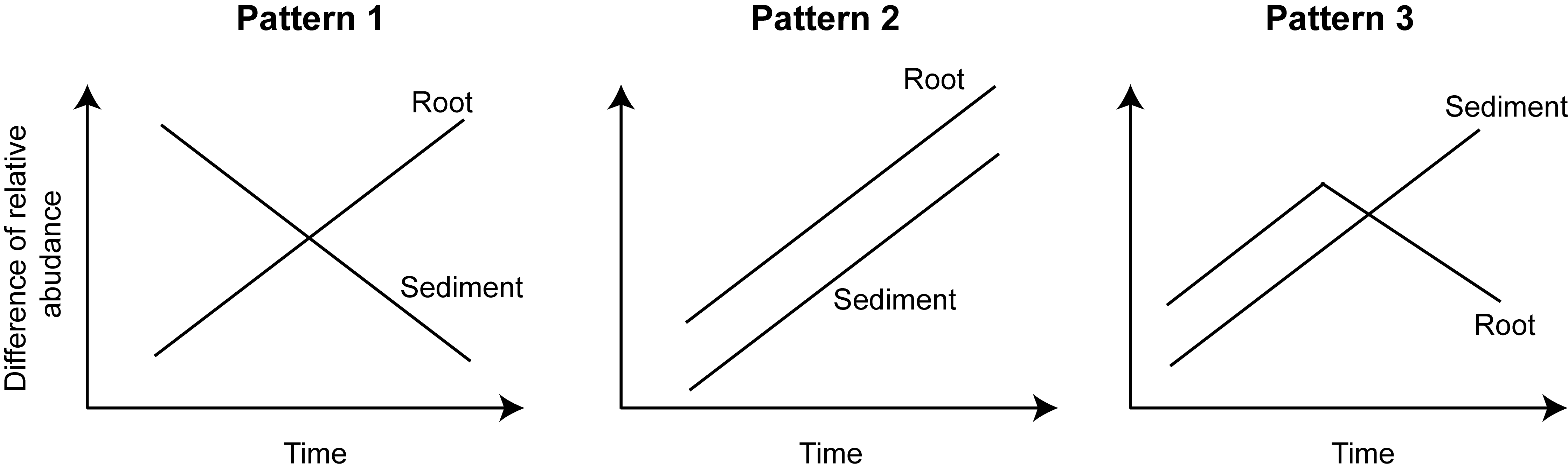


**Fig. S9.** Abundance variation trend of different subgroups along with temporal variation.

**Table S1.** ASV number and sequence number of different subgroups.

|  |  | ASV Number | | | | |  | | Sequence Number | | | | | |  |
| --- | --- | --- | --- | --- | --- | --- | --- | --- | --- | --- | --- | --- | --- | --- | --- |
|  |  | Total |  | Control |  | Exp | |  | | Total |  | Control |  | Exp | |
| Rhizosphere |  | 7198 |  | 6799 |  | 6542 | |  | | 218232 |  | 124704 |  | 93528 | |
| Sediment | | 6206 |  | 5282 |  | 5392 | |  | | 187056 |  | 93528 |  | 93528 | |
| All |  | 7357 |  | 7071 |  | 6902 | |  | | 405288 |  | 218232 |  | 187056 | |

**Table S2.** Nitrogen cycling genes of KEGG pathways related to nitrogen metabolism (KO00910).

| **KO** | **Gene** | **Function** |
| --- | --- | --- |
| nifD | K02586 | Nitrogen fixation |
| nifH | K02588 | Nitrogen fixation |
| nifK | K02591 | Nitrogen fixation |
| narG | K00370 | Denitrification and dissimilatory nitrate reduction |
| nrxA | K00370 | Nitrification |
| narH | K00371 | Denitrification and dissimilatory nitrate reduction |
| nrxB | K00371 | Nitrification |
| narI | K00374 | Denitrification and dissimilatory nitrate reduction |
| napA | K02567 | Denitrification and dissimilatory nitrate reduction |
| napB | K02568 | Denitrification and dissimilatory nitrate reduction |
| nirB | K00362 | Dissimilatory nitrate reduction |
| nirD | K00363 | Dissimilatory nitrate reduction |
| nrfA | K03385 | Dissimilatory nitrate reduction |
| nrfH | K15876 | Dissimilatory nitrate reduction |
| narB | K00367 | Assimilatory nitrate reduction |
| nasA | K00372 | Assimilatory nitrate reduction |
| nirA | K00366 | Assimilatory nitrate reduction |
| nirS | K00368 | Denitrification |
| nirK | K15864 | Denitrification |
| norC | K02305 | Denitrification |
| norB | K04561 | Denitrification |
| nosZ | K00376 | Denitrification |

**Table S3.** Topological characteristics of networks.

|  | Clustering coefficient | Number of Nodes | Network density | Network diameter | Network heterogeneity | Network centralization | Characteristic  path distance | Average  Degree |
| --- | --- | --- | --- | --- | --- | --- | --- | --- |
| RSC | 0.432 | 180 | 0.079 | 9 | 0.827 | 0.185 | 3.129 | 14.211 |
| RSB | 0.297 | 158 | 0.023 | 15 | 0.560 | 0.041 | 5.891 | 3.671 |
| SC | 0.376 | 187 | 0.033 | 10 | 0.741 | 0.076 | 4.264 | 6.096 |
| SB | 0.374 | 174 | 0.036 | 12 | 0.900 | 0.115 | 4.649 | 6.287 |

**Table S4.** Keystone taxa in four groups.

| **RSC** | **Phylum** | **Class** | **Order** | **Family** | **Genus** | **Species** |
| --- | --- | --- | --- | --- | --- | --- |
| ASV_6723 | Bacteroidetes | Unclassified | Unclassified | Unclassified | Unclassified | Unclassified |
| ASV_3747 | Proteobacteria | Deltaproteobacteria | Desulfobacterales | Desulfobacteraceae | Desulfosarcina | Unclassified |
| ASV_6580 | Proteobacteria | Deltaproteobacteria | Desulfobacterales | Desulfobacteraceae | Unclassified | Unclassified |
| ASV_3569 | Proteobacteria | Epsilonproteobacteria | Campylobacterales | Helicobacteraceae | Sulfurimonas | Unclassified |
| ASV_7042 | Proteobacteria | Gammaproteobacteria | Unclassified | Unclassified | Unclassified | Unclassified |
| ASV_5640 | Proteobacteria | Gammaproteobacteria | Chromatiales | Unclassified | Unclassified | Unclassified |
| ASV_5132 | Proteobacteria | Deltaproteobacteria | Desulfobacterales | Desulfobacteraceae | Unclassified | Unclassified |
| ASV_6706 | Proteobacteria | Deltaproteobacteria | Desulfobacterales | Desulfobacteraceae | Unclassified | Unclassified |
| ASV_7104 | Proteobacteria | Deltaproteobacteria | Desulfobacterales | Desulfobacteraceae | Unclassified | Unclassified |
| ASV_2410 | Proteobacteria | Gammaproteobacteria | Chromatiales | Unclassified | Unclassified | Unclassified |
| ASV_6917 | Proteobacteria | Deltaproteobacteria | Desulfobacterales | Desulfobacteraceae | Unclassified | Unclassified |
| ASV_7084 | Actinobacteria | Actinobacteria | Actinomycetales | Unclassified | Unclassified | Unclassified |
| ASV_2312 | Proteobacteria | Deltaproteobacteria | Desulfobacterales | Desulfobacteraceae | Desulfosarcina | Unclassified |
| ASV_4849 | Proteobacteria | Gammaproteobacteria | Unclassified | Unclassified | Unclassified | Unclassified |
| ASV_4005 | Proteobacteria | Deltaproteobacteria | Desulfobacterales | Desulfobulbaceae | Desulfobulbus | Desulfobulbus_rhabdoformis |
| ASV_7650 | Proteobacteria | Deltaproteobacteria | Desulfobacterales | Desulfobacteraceae | Unclassified | Unclassified |
| ASV_4993 | Proteobacteria | Deltaproteobacteria | Desulfobacterales | Desulfobacteraceae | Unclassified | Unclassified |
| ASV_1860 | Proteobacteria | Deltaproteobacteria | Myxococcales | Kofleriaceae | Kofleria | Kofleria_flava |
| ASV_3185 | Spirochaetes | Spirochaetia | Spirochaetales | Spirochaetaceae | Spirochaeta | Unclassified |
| ASV_2803 | Proteobacteria | Gammaproteobacteria | Unclassified | Unclassified | Unclassified | Unclassified |
| ASV_4926 | Proteobacteria | Gammaproteobacteria | Unclassified | Unclassified | Unclassified | Unclassified |
| ASV_6149 | Proteobacteria | Deltaproteobacteria | Desulfobacterales | Desulfobacteraceae | Unclassified | Unclassified |
| ASV_6984 | Proteobacteria | Deltaproteobacteria | Desulfobacterales | Desulfobacteraceae | Unclassified | Unclassified |
| ASV_7273 | Unclassified | Unclassified | Unclassified | Unclassified | Unclassified | Unclassified |
| ASV_7257 | Unclassified | Unclassified | Unclassified | Unclassified | Unclassified | Unclassified |
| ASV_3368 | Proteobacteria | Gammaproteobacteria | Unclassified | Unclassified | Unclassified | Unclassified |
| ASV_2572 | Proteobacteria | Deltaproteobacteria | Desulfobacterales | Desulfobacteraceae | Unclassified | Unclassified |
| ASV_3984 | Actinobacteria | Actinobacteria | Actinomycetales | Unclassified | Unclassified | Unclassified |
| ASV_5053 | Unclassified | Unclassified | Unclassified | Unclassified | Unclassified | Unclassified |

| **RSB** | **Phylum** | **Class** | **Order** | **Family** | **Genus** | **Species** |
| --- | --- | --- | --- | --- | --- | --- |
| ASV_5774 | Proteobacteria | Gammaproteobacteria | Thiotrichales | Piscirickettsiaceae | Methylophaga | Unclassified |
| ASV_7650 | Proteobacteria | Deltaproteobacteria | Desulfobacterales | Desulfobacteraceae | Unclassified | Unclassified |
| ASV_7116 | Bacteroidetes | Unclassified | Unclassified | Unclassified | Unclassified | Unclassified |
| ASV_7448 | Firmicutes | Unclassified | Unclassified | Unclassified | Unclassified | Unclassified |
| ASV_7603 | Cyanobacteria | Chloroplast | f__Chloroplast | g__Bacillariophyta | Unclassified | Unclassified |
| ASV_7641 | Proteobacteria | Alphaproteobacteria | Rhodobacterales | Rhodobacteraceae | Unclassified | Unclassified |
| ASV_4026 | Proteobacteria | Alphaproteobacteria | Unclassified | Unclassified | Unclassified | Unclassified |
| ASV_4145 | Proteobacteria | Gammaproteobacteria | Chromatiales | Unclassified | Unclassified | Unclassified |
| ASV_7676 | Deferribacteres | Deferribacteres | Deferribacterales | Deferribacterales_incertae_sedis | Caldithrix | Unclassified |
| ASV_3899 | Proteobacteria | Deltaproteobacteria | Desulfobacterales | Desulfobacteraceae | Unclassified | Unclassified |
| ASV_4993 | Proteobacteria | Deltaproteobacteria | Desulfobacterales | Desulfobacteraceae | Unclassified | Unclassified |
| ASV_2506 | Chloroflexi | Unclassified | Unclassified | Unclassified | Unclassified | Unclassified |
| ASV_6137 | Proteobacteria | Deltaproteobacteria | Desulfobacterales | Desulfobacteraceae | Unclassified | Unclassified |
| ASV_4197 | Proteobacteria | Deltaproteobacteria | Desulfobacterales | Desulfobacteraceae | Desulfosarcina | Unclassified |

| **SC** | **Phylum** | **Class** | **Order** | **Family** | **Genus** | **Species** |
| --- | --- | --- | --- | --- | --- | --- |
| ASV_5774 | Proteobacteria | Gammaproteobacteria | Thiotrichales | Piscirickettsiaceae | Methylophaga | Unclassified |
| ASV_7605 | Proteobacteria | Alphaproteobacteria | Rhodobacterales | Rhodobacteraceae | Unclassified | Unclassified |
| ASV_6483 | Bacteroidetes | Bacteroidia | Bacteroidales | Marinilabiliaceae | Carboxylicivirga | Carboxylicivirga_taeanensis |
| ASV_2605 | Proteobacteria | Epsilonproteobacteria | Campylobacterales | Helicobacteraceae | Sulfurovum | Sulfurovum_aggregans |
| ASV_5340 | Proteobacteria | Alphaproteobacteria | Rhizobiales | Unclassified | Unclassified | Unclassified |
| ASV_6641 | Proteobacteria | Deltaproteobacteria | Desulfobacterales | Desulfobacteraceae | Unclassified | Unclassified |
| ASV_7273 | Unclassified | Unclassified | Unclassified | Unclassified | Unclassified | Unclassified |
| ASV_6011 | Proteobacteria | Alphaproteobacteria | Rhodobacterales | Rhodobacteraceae | Unclassified | Unclassified |
| ASV_6927 | Latescibacteria | Latescibacteria_genera_incertae_sedis | Unclassified | Unclassified | Unclassified | Unclassified |
| ASV_5556 | Proteobacteria | Deltaproteobacteria | Desulfobacterales | Desulfobacteraceae | Unclassified | Unclassified |
| ASV_4171 | Proteobacteria | Deltaproteobacteria | Desulfobacterales | Desulfobacteraceae | Unclassified | Unclassified |
| ASV_7116 | Bacteroidetes | Unclassified | Unclassified | Unclassified | Unclassified | Unclassified |
| ASV_7292 | Firmicutes | Clostridia | Clostridiales | Ruminococcaceae | Unclassified | Unclassified |
| ASV_6796 | Cyanobacteria | Chloroplast | Chloroplast | Bacillariophyta | Unclassified | Unclassified |
| ASV_5966 | Proteobacteria | Deltaproteobacteria | Desulfobacterales | Desulfobacteraceae | Unclassified | Unclassified |
| ASV_6601 | Bacteroidetes | Unclassified | Unclassified | Unclassified | Unclassified | Unclassified |
| ASV_6814 | Proteobacteria | Alphaproteobacteria | Rhodobacterales | Rhodobacteraceae | Ruegeria | Ruegeria_arenilitoris |
| ASV_6050 | Bacteroidetes | Bacteroidia | Bacteroidales | Unclassified | Unclassified | Unclassified |
| ASV_3569 | Proteobacteria | Epsilonproteobacteria | Campylobacterales | Helicobacteraceae | Sulfurimonas | Unclassified |
| ASV_2456 | Proteobacteria | Gammaproteobacteria | Unclassified | Unclassified | Unclassified | Unclassified |
| ASV_4110 | Actinobacteria | Unclassified | Unclassified | Unclassified | Unclassified | Unclassified |
| ASV_2466 | Proteobacteria | Deltaproteobacteria | Desulfobacterales | Desulfobacteraceae | Unclassified | Unclassified |
| ASV_6723 | Bacteroidetes | Unclassified | Unclassified | Unclassified | Unclassified | Unclassified |
| ASV_6039 | Bacteroidetes | Bacteroidia | Bacteroidales | Unclassified | Unclassified | Unclassified |
| ASV_4043 | Deferribacteres | Deferribacteres | Deferribacterales | Deferribacterales_incertae_sedis | Caldithrix | Caldithrix_palaeochoryensis |
| ASV_7384 | Bacteroidetes | Flavobacteriia | Flavobacteriales | Flavobacteriaceae | Actibacter | Actibacter_sediminis |
| ASV_4431 | Proteobacteria | Deltaproteobacteria | Desulfobacterales | Desulfobacteraceae | Unclassified | Unclassified |
| ASV_6920 | Actinobacteria | Actinobacteria | Unclassified | Unclassified | Unclassified | Unclassified |
| ASV_2445 | Firmicutes | Clostridia | Clostridiales | Peptostreptococcaceae | Clostridium_XI | Clostridium_litorale |
| ASV_1169 | Bacteroidetes | Flavobacteriia | Flavobacteriales | Flavobacteriaceae | Unclassified | Unclassified |
| ASV_6861 | Deferribacteres | Deferribacteres | Deferribacterales | Deferribacterales_incertae_sedis | Caldithrix | Caldithrix_palaeochoryensis |

| **SB** | **Phylum** | **Class** | **Order** | **Family** | **Genus** | **Species** |
| --- | --- | --- | --- | --- | --- | --- |
| ASV_5891 | Unclassified | Unclassified | Unclassified | Unclassified | Unclassified | Unclassified |
| ASV_2097 | Proteobacteria | Gammaproteobacteria | Chromatiales | Unclassified | Unclassified | Unclassified |
| ASV_1337 | Proteobacteria | Deltaproteobacteria | Desulfobacterales | Desulfobacteraceae | Unclassified | Unclassified |
| ASV_1972 | Proteobacteria | Gammaproteobacteria | Chromatiales | Chromatiaceae | Unclassified | Unclassified |
| ASV_4849 | Proteobacteria | Gammaproteobacteria | Unclassified | Unclassified | Unclassified | Unclassified |
| ASV_2493 | Proteobacteria | Gammaproteobacteria | Chromatiales | Unclassified | Unclassified | Unclassified |
| ASV_7373 | Acidobacteria | Acidobacteria_Gp22 | g__Gp22 | Unclassified | Unclassified | Unclassified |
| ASV_7092 | Bacteroidetes | Flavobacteriia | Flavobacteriales | Flavobacteriaceae | Unclassified | Unclassified |
| ASV_7185 | Unclassified | Unclassified | Unclassified | Unclassified | Unclassified | Unclassified |
| ASV_3899 | Proteobacteria | Deltaproteobacteria | Desulfobacterales | Desulfobacteraceae | Unclassified | Unclassified |
| ASV_2732 | Proteobacteria | Gammaproteobacteria | Unclassified | Unclassified | Unclassified | Unclassified |
| ASV_3956 | Proteobacteria | Deltaproteobacteria | Desulfobacterales | Desulfobacteraceae | Unclassified | Unclassified |
| ASV_7084 | Actinobacteria | Actinobacteria | Actinomycetales | Unclassified | Unclassified | Unclassified |
| ASV_3984 | Actinobacteria | Actinobacteria | Actinomycetales | Unclassified | Unclassified | Unclassified |
| ASV_7461 | Proteobacteria | Epsilonproteobacteria | Campylobacterales | Helicobacteraceae | Sulfurovum | Sulfurovum_aggregans |
| ASV_5011 | Proteobacteria | Alphaproteobacteria | Rhodobacterales | Rhodobacteraceae | Unclassified | Unclassified |
| ASV_6456 | Proteobacteria | Gammaproteobacteria | Chromatiales | Unclassified | Unclassified | Unclassified |
| ASV_6984 | Proteobacteria | Deltaproteobacteria | Desulfobacterales | Desulfobacteraceae | Unclassified | Unclassified |
| ASV_6858 | Actinobacteria | Actinobacteria | Actinomycetales | Unclassified | Unclassified | Unclassified |
| ASV_6050 | Bacteroidetes | Bacteroidia | Bacteroidales | Unclassified | Unclassified | Unclassified |
| ASV_3228 | Proteobacteria | Deltaproteobacteria | Desulfobacterales | Desulfobulbaceae | Unclassified | Unclassified |
| ASV_6824 | Fusobacteria | Fusobacteriia | Fusobacteriales | Fusobacteriaceae | Propionigenium | Propionigenium_maris |
| ASV_3890 | Proteobacteria | Gammaproteobacteria | Chromatiales | Unclassified | Unclassified | Unclassified |
| ASV_7061 | Proteobacteria | Deltaproteobacteria | Unclassified | Unclassified | Unclassified | Unclassified |
| ASV_6503 | Deferribacteres | Deferribacteres | Deferribacterales | Deferribacterales_incertae_sedis | Caldithrix | Caldithrix_palaeochoryensis |
